# Phonon–Phonon Interactions in the Polarization Dependence of Raman Scattering

**DOI:** 10.1021/acs.jpcc.3c03850

**Published:** 2023-08-31

**Authors:** Nimrod Benshalom, Maor Asher, Rémy Jouclas, Roman Korobko, Guillaume Schweicher, Jie Liu, Yves Geerts, Olle Hellman, Omer Yaffe

**Affiliations:** † Department of Chemical and Biological Physics, Weizmann Institute of Science, Rehovot 76100, Israel; ‡ Laboratoire de Chimie des Polymères, Universit́e Libre de Bruxelles (ULB), Brussels 1050, Belgium; § International Solvay Institutes for Physics and Chemistry, Brussels 1050, Belgium; ∥ Department of Physics, Chemistry and Biology (IFM), Linköping University, Linköping SE-581 83, Sweden; ⊥ Department of Molecular Chemistry and Material Science, Weizmann Institute of Science, Rehovot 76100, Israel

## Abstract

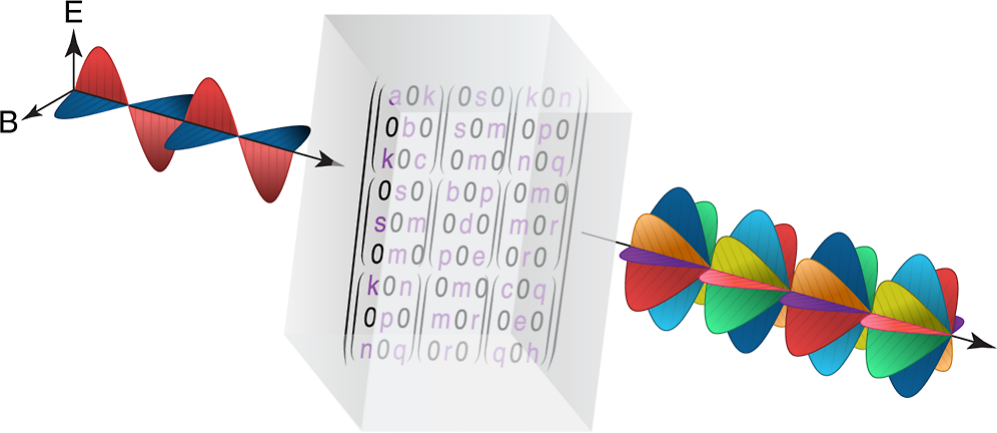

We have found that the polarization dependence of Raman scattering in organic crystals at finite temperatures can only be described by a fourth-rank tensor formalism. This generalization of the second-rank Raman tensor  stems from the effect of off-diagonal components in the crystal self-energy on the light scattering mechanism. We thus establish a novel manifestation of phonon–phonon interaction in inelastic light scattering, markedly separate from the better-known phonon lifetime.

## 1 Introduction

**Figure 1:**
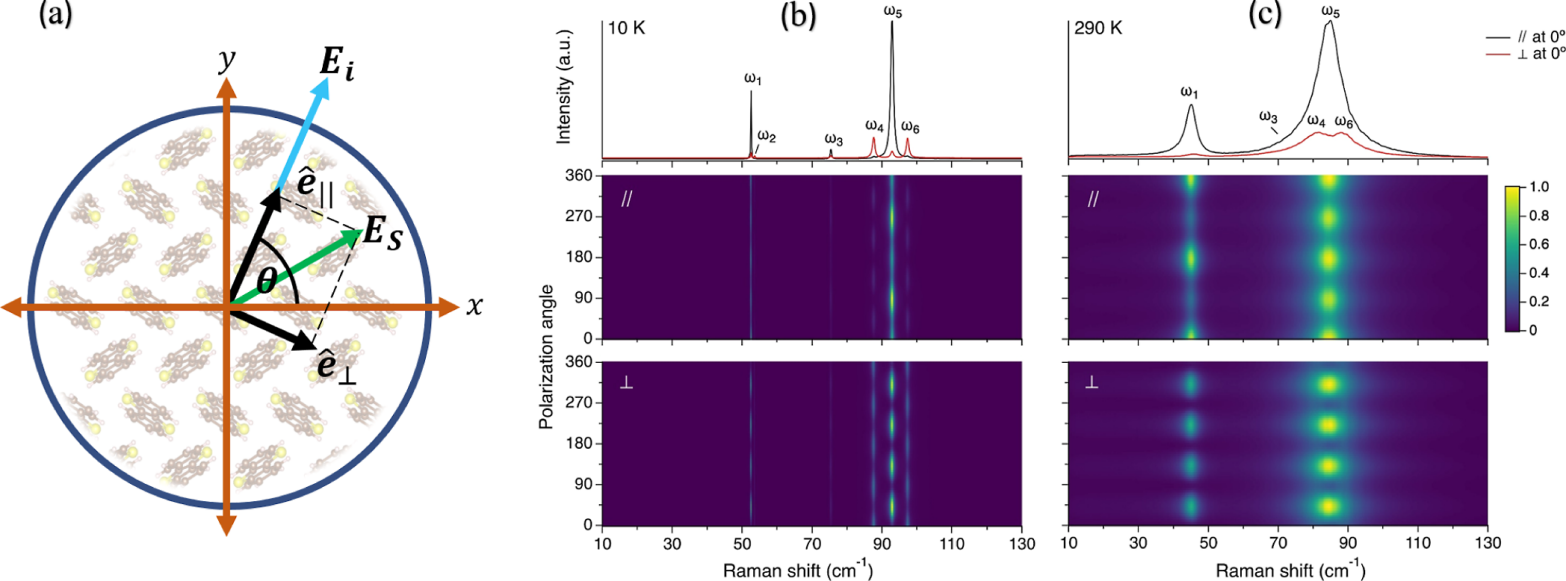
(a) Face-on schematic of a PO measurement. (b) PO measurement of BTBT at 10 K. (c) PO measurement of BTBT at 290 K. An example spectrum (θ = 0°) for each configuration is given (top panels). Parallel measurement probes the scattered field’s projection on  (middle panels), and cross measurement probes the scattered field’s projection on  (bottom panels).

The Raman spectrum of crystals is usually interpreted through the prism of mode assignment, where each spectral peak is assigned an irreducible representation (irrep) of the relevant space group.^1^ So entrenched was this perspective that past studies would denote their measurement geometry in terms of the irreps observable by it.^2−8^ This conceptual framework, also known as factor group analysis, provides a rigorous tool set to connect observed spectra with crystal structure and vibrational symmetries. Besides predicting the number of Raman active modes and their degeneracy, factor group analysis also dictates the polarization dependence of a spectral feature associated with a specific irrep. The connection between the polarization of incident and scattered light is thus governed by the assigned irrep. Most textbooks,^9−14^ as well as contemporary research,^15−18^ use the matrix representation, also known as the Raman tensor, to describe this irrep-specific polarization dependence. In this matrix formalism, the vector-dependent scattering cross-section of a Raman peak assigned an irrep Γ_*x,*_ and the corresponding Raman tensor , is described by^9^
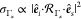
1where  and  are the incident and collected light polarization unit vectors, respectively. This expression completely governs the polarization dependence of the Raman signal, with the angle entering through the geometrical relationship between the polarization unit vectors and crystal orientation (see Figure 1a). The components of  are contracted susceptibility derivatives evaluated at equilibrium, so along with its persistent irrep identification, eq 1 predicts a temperature-independent polarization pattern.

However, some of us recently showed that the polarization–orientation (PO) Raman response, i.e., the polarization dependence of the Raman signal, evolves with temperature for some organic crystals.^19,20^ These kinds of PO patterns and their temperature dependence cannot be captured by any superposition of irrep-specific matrices, and a generalization to a fourth-rank formalism is necessary. We argue this is the result of specific phonon–phonon interactions entering the Raman scattering process, an effect which is highly temperature and material-dependent. To further elucidate the physical origins of the effect, we adopt a generalized many-body treatment of the Raman scattering cross-section. We find that by allowing off-diagonal components in the self-energy to persist, different irrep contributions in the spectrum are mixed, and a fourth-rank formalism is engendered. Because the magnitude of these components is expected to be temperature-dependent, this accounts for both the observed PO pattern and its temperature evolution. We confirm the validity of the model by successfully fitting it to experimental spectra.

This inadequacy of eq 1 stems from the naïve treatment of temperature dependence in its derivation, which implicitly assumes a constant parabolic potential surface for atomic displacements, also known as the harmonic approximation.^9,21^ The role of anharmonicity in solid-state Raman spectroscopy has been the subject of extensive study, not only from an analytical point of view,^22−24^ but also from computational,^25−27^ and experimental^28−32^ perspectives. However, despite the consensus over the prominence of anharmonicity in Raman scattering, its effect on polarization has not been considered before Asher et al.^19^

We apply our generalized model on the PO spectra of a few organic crystals, focusing on [1]benzothieno[3,2-*b*]benzothiophene (BTBT) as an ideal showcase. As promising organic semiconducting molecular crystals, BTBT and its derivatives have been intensively investigated due to their high mobility and other application-oriented properties, such as air-stability and solution processability.^33−38^ Besides its scientific importance, BTBT proved especially suitable for this study as its separate spectral features display the different possible PO trends with temperature, showing that the need for a fourth-rank formalism is not only material and temperature dependent but may also be mode-specific.

## 2 Polarization Dependence of the Raman Spectrum of BTBT

Our experimental setup employed a back-scattering geometry, as shown in Figure 1a. In a PO measurement, the polarization of the incident laser beam (***E***_*i*_) is rotated in a plane parallel to the BTBT (001) crystal face. The inelastically back-scattered light can generally assume any polarization (***E***_*s*_). We separately probe the projection of scattered light onto either a parallel  or cross  polarization with respect to the incident beam. Full details for the measurement procedure and sample characterization are given in sections SI-II in the Supporting Information.^39−43^

Figure 1b,c presents the Stokes Raman PO data for BTBT measured at 10 and 290 K, respectively. At 10 K, we observe exactly six sharp peaks, as predicted by Raman selection rules (see section SIX.1 in the Supporting Information(44)). At 290 K, we observe five distinguishable peaks due to the low intensity of ω_2_ and the broadness of the spectrum. The correspondence between spectral peaks across temperatures is corroborated by continuously sampling the Raman spectra throughout the temperature range (section SVI in the Supporting Information).

The polarization angle (vertical axis) in Figure 1b,c is off-set by a random angle controlled by the crystal orientation in the laboratory frame but is consistent for all spectra.

To examine the PO dependence of each spectral feature, we must first extract its integrated intensity. We do this by fitting the raw spectra to a sum of six Lorentzians, in agreement with factor group analysis, and then extracting the integrated intensity of each separate peak (see section SIII in the Supporting Information). Because we are only interested in the polarization dependence, other deconvolution procedures may be used. We perform this process for the PO measurements at 10, 80, 150, 220, and 290 K (see sections SVII and SVIII in Supporting Information for the raw data and extracted temperature-dependent PO pattern of each spectral feature, respectively). With the PO pattern at hand, we can apply our model of choice to interpret it.

**Figure 2:**
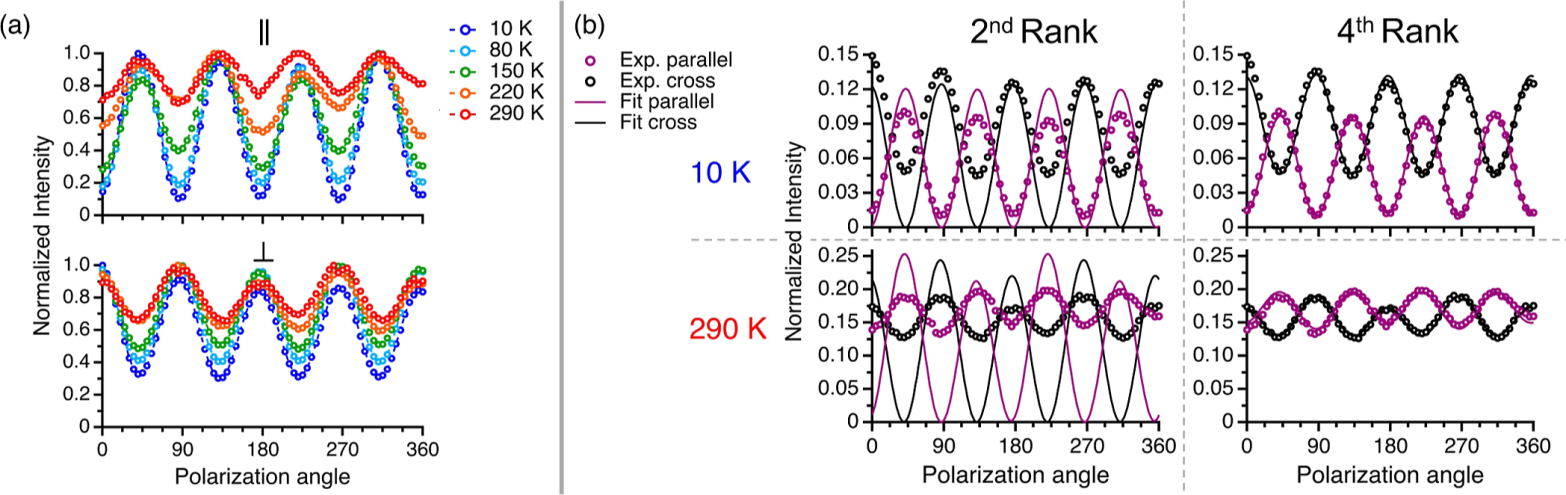
(a) PO Raman response of ω_4_ from 10 to 290 K in parallel (top panel) and cross (bottom panel) configurations. (b) Fit results of the PO Raman response of ω_4_ at 10 K (top panels) and 290 K (bottom panels) using the second-rank (left panels) and fourth-rank Raman tensor formalism (right panels).

Figure 2a presents the temperature evolution of the PO pattern for peak ω_4_ extracted from Figure 1, i.e., the change with temperature of the polarization dependence of its integrated intensity. The PO pattern is highly temperature dependent in both parallel and cross configurations (see section SIV in Supporting Information for the temperature-independent PO response of silicon^45^). This temperature dependence in BTBT is fully reversible upon cooling, ruling out static disorder as its origin.

Following the harmonic approximation, we apply eq 1 and determine the appropriate irrep Γ_*x*_ and its corresponding Raman matrix . We account for birefringence effects by using a corrected Raman tensor, introducing a relative phase factor between the diagonal tensor components of .^17^ For our alternative, temperature-dependent treatment, we follow Cowley^46^ and Born and Huang^47^ before him, who described the Raman cross-section with a fourth-rank tensor
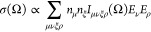
2where Ω is the probing frequency, ***E*** is the incident electric field, and ***n*** is the unit vector for the observed polarization, with Greek letters standing for Cartesian components. Because the Raman matrix in eq 1 is squared, casting it into a fourth-ranked tensor is immediately valid. However, its necessity compared to the prevalent second-rank formalism has never been demonstrated experimentally, nor has it been considered theoretically. This approach assumes only a symmetry-constrained fourth-rank tensor without invoking any specific scattering mechanism. We discuss the physical justification for eq 2 below, but for now treat *I*_μνξρ_ phenomenologically as the general material property governing light scattering in the crystal. The tensor structure and component ratios of *I*_μνξρ_ will determine the PO pattern observed in experiment. Unlike eq 1, this generalized form has the potential of producing non-negative values, but this is irrelevant when fitting experimental data to eq 2. We discuss the positive semi-definiteness of the scattering tensor in section SIX. 5 in the Supporting Information.^48−50^

Figure 2b shows the fitting attempts using both the predicted irrep (left panels) and generalized fourth-rank tensor (right panels) for the same ω_4_ spectral feature at 10 and 290 K (see sections SIX.2-3 in Supporting Information for all fitting tensors and results^9,17,19,51^). Using the harmonic Raman tensor, we find a partial fit at 10 K, which gradually deteriorates as temperature increases up to room temperature. In stark contrast, the fourth-rank tensor formalism gives a near perfect fit for all data sets. A general fourth-rank tensor obviously offers a much larger parameter space, but symmetry considerations dramatically reduce the number of independent variables in *I*_μνξρ_. We thus find the correct observable material property governing the Raman scattering around ω_4_ is a fourth-rank tensor. Similar behavior was previously observed in the Raman spectra of anthracene and pentacene crystals,^19^ where again, the appropriate fourth-rank tensor faithfully reproduces all PO patterns (see section SX in the Supporting Information(19)).

The matrix  used in Figure 2b is the specific irrep appropriate one. However, even a completely unconstrained second-rank tensor, with an additional phase parameter for birefringence effects,^17^ was not able to successfully fit eq 1. Importantly, in our experiment, this generalized form for  has more parameters than the symmetry-reduced form of *I*_μνξρ_ (five vs four). The different PO patterns made possible by each formalism depend on the measuring geometry, but one concrete example is demonstrated in Figure 2; in our back-scattering setup, any symmetric second-rank tensor will make the cross signal vanish with fourfold periodicity. Indeed, this prohibits a successful fit to many of the observed spectra. Conversely, the fourth-rank tensor has no such limitation, allowing a faithful reproduction of the measured PO patterns.

## 3 Fourth-Rank Tensor Description of Raman Scattering

### 3.1 Theory of Inelastic Light Scattering at Finite Temperatures

We reiterate the tenets of the theory of inelastic light scattering from crystals at finite temperatures for clarity and describe the novel aspect regarding polarization dependence demonstrated in this study.

Assuming only dipole scattering, the inelastic light scattering of a crystal as a function of probing frequency Ω is governed by^46^
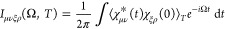
3the Fourier transform of the thermally averaged susceptibility auto-correlation function at temperature *T*. The presentation here and full derivation in section SXI of the Supporting Information use Hartee atomic units.^21,27,52−58^ The fundamental physical entity—susceptibility (**χ**), is indeed a second-rank tensor operator but since experimentally we measure intensity, the observable is defined by the tensor product of two susceptibility operators.

Using normal mode coordinates *A*_**q**s_ for mode s with momentum **q** and expanding the susceptibility in atomic displacements to first order, eq 3 transforms to^59^
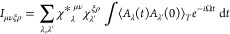
4where we adopted a composite index λ to denote ***q****s*, and χ_λ_^μν^ are the susceptibility derivatives with respect to normal-mode displacement λ. The thermal average ⟨···⟩_*T*_ in eq 4 is taken over crystal configurations. Except for the susceptibility derivatives, previously discussed by some of us,^27^ the problem has been transformed to one of lattice dynamics. Under the harmonic approximation, the double sum in eq 4 reduces to a single sum over normal modes λ, and the matrix nature of eq 1 is recovered (see explicit calculation in section XI.3 of the Supporting Information).

The ionic motion inside a crystal can be solved with the single-particle Green’s function *G*(Ω),^56^ commonly written as a sum of the harmonic Green’s function *g*_λ_^0^ and a complex self-energy term Σ_λλ′_
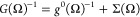
5We can make eq 4 more analytically transparent by using the corresponding spectral function^56^
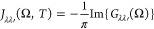
6so the generalized scattering tensor becomes
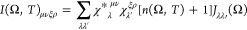
7where *n*(Ω, *T*) is the Bose–Einstein occupation number for energy ℏΩ at temperature *T*. Note that eq 7 is equivalent to eq 4; the reformulation only serves as a means to introduce anharmonic effects (see section SXI.2 in Supporting Information for the detailed derivation).

### 3.2 Two-Mode Model for Inelastic Light Scattering

To demonstrate how phonon–phonon interactions lead to a fourth-rank tensor description, we evaluate eq 7 for the simplest case of two “mixed” normal modes with different irreps. The derivatives  are the same as in the harmonic treatment, so the Raman selection rules remain valid. The BTBT structure only allows for A_g_ and B_g_ Raman active modes.^44^ However, these selection rules do not prohibit multiple terms from contributing to the same frequency interval. Retaining all Raman active modes will yield an intractable expression, so we consider the mixing of two modes with different irreps. Absorbing the diagonal self-energy real part into the resonance frequencies ω_*s*_, our harmonic Green’s function and self-energy term become^56^
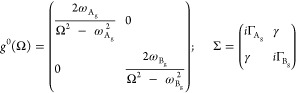
8where we assumed real off-diagonal self-energy components γ, originally introduced by Barker and Hopfield to explain the infrared reflectivity of perovskites,^60^ later used to explain their Raman spectra,^61,62^ but hitherto ignored in vector (polarization) analysis. Importantly, these past studies invoked γ to explain spectral line shapes, and are therefore open to competing diagonal self-energy descriptions. Because our PO measurements probe the tensor structure of the scattering mechanism, we are able to exclude a diagonal model and thus demonstrate the unique signature of off-diagonal self-energy components. Generally, the self-energy is also frequency dependent, Σ = Σ(Ω), but since the fitted spectral features are relatively sharp (3 cm^–1^), we assume a constant. The full spectral function, (eq S11), is given in Sec. SXI.4 in the Supporting Information.

The mixed derivative terms  vanish due to symmetry considerations. By explicitly rewriting the tensorial part of eq 7 for our two-mode model, we obtain
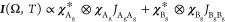
9It would appear that we have recovered a quasi-harmonic expression, with each term corresponding to a separate spectral feature. However, the off-diagonal component γ dramatically changes the frequency dependence of the spectral functions *J*(Ω). Instead of a single Lorentzian-like feature centered around a single resonance frequency, they now each have appreciable contributions in both  and . It is impossible to re-create the tensorial structure of eq 9 by squaring a single matrix (eq 1), and a fourth-rank tensor formalism must be invoked.

Another way to understand the unique effect of the off-diagonal components is to examine the temperature dependence of the phonon’s eigenvectors. Whereas diagonal self-energy components modify the phonon’s lifetime and resonance frequency (i.e., eigenvalue), the off-diagonal components change the eigenvector, or identity, of the vibrational mode. We can parameterize the linear combination describing the re-normalized eigenvectors using the self-energy term. If the temperature-dependent spectral function is diagonalized by
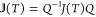
10then the temperature-dependent eigenvector ***v***_*i*_(*T*) is given by
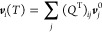
11where the index *j* sums over all the zero-temperature (harmonic) eigenvectors ***v***_*j*_^0^ within the coupled subspace (two in our case), and the dimensionality of ***v***_*i*_ is determined, as usual, by the number of atoms in the unit cell.

**Figure 3:**
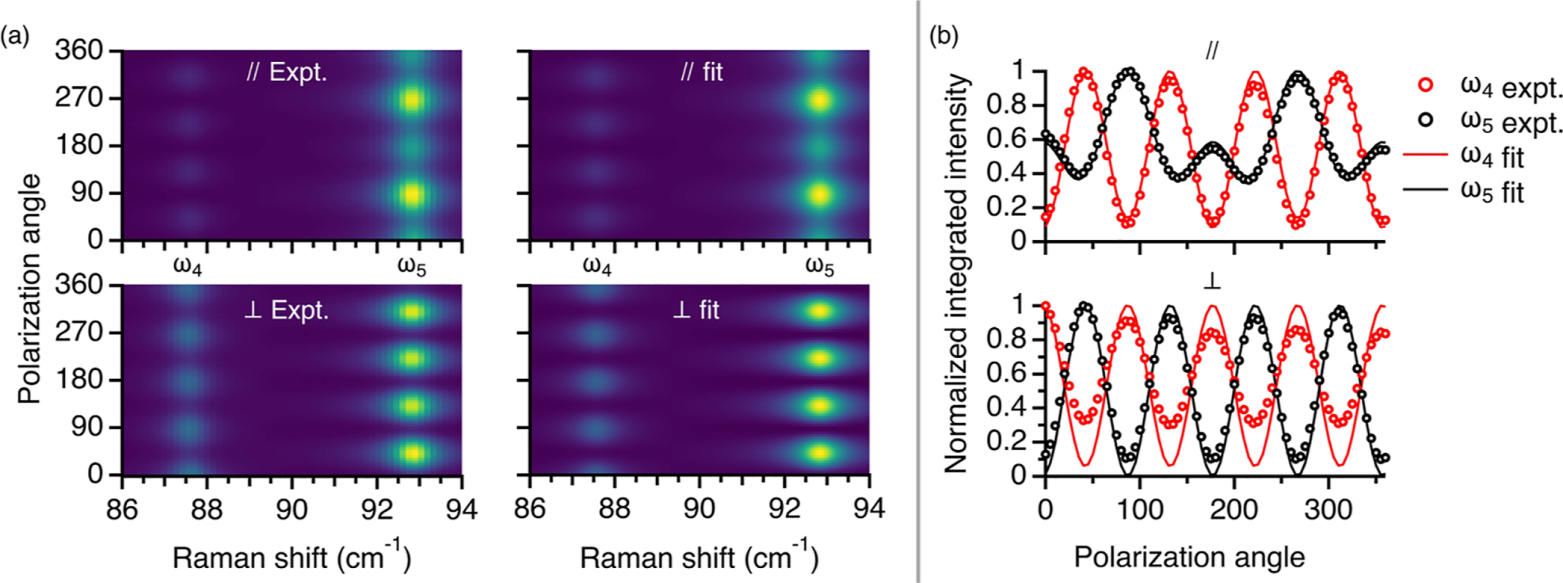
Global fit of the two-mode model for the PO patterns of spectral features ω_4_ and ω_5_ in the Raman spectra of BTBT. (a) Measurement (left) and fit (right) for parallel (top) and cross (bottom) configurations. (b) Integrated intensity of each feature as extracted from measurement (circles) and fit (lines).

The model described by eqs 2 and 9 goes beyond the phenomelogical treatment described in Section 2 by adopting a specific scattering mechanism, namely, a Taylor expansion of the susceptibility in atomic displacements around the equilibrium crystal structure. It is tested by performing a global fit to the measured spectral range around ω_5_ (A_g_) and ω_4_ (B_g_) in BTBT (Figure 3). Besides their appropriate irreps, our choice of peaks is guided by the PO pattern of ω_4_ that defies eq 1, and by their spectral proximity, predicted to increase the effect of mixing between the spectral functions (see eq S11 in section SIX.4 in Supporting Information).

The spectra for a full set of PO measurements (parallel and cross) at a given temperature (*T* = 10 *K*) are fitted simultaneously to the two-mode model. All fit functions, parameters, and procedures are given in section SIX.4 in the Supporting Information. Figure 3a presents the zoomed-in PO measurement at 10 K alongside the fit results for the same frequency range. The integrated intensity of each feature in each configuration was extracted from the fit and compared to the experimental values in Figure 3b. We find the parallel signal (top) matches the fit perfectly, with the cross (bottom) showing a slightly exaggerated oscillation amplitude, especially for ω_4_.

The global fit uses five parameters for all spectra and is therefore not as accommodating as the fully general fourth-rank tensor used in Figure 2, where the PO of each peak is independently fitted with four parameters. The pertinent comparison, however, is to the original harmonic model of eq 1. Even when fully relaxing symmetry constraints and allowing for birefringence (totaling in seven independent parameters), eq 1 gave an inferior fit compared to the two-mode model. We emphasize that although all equations are strictly rigorous, substantial simplifying approximations were introduced into the model. Our choice to mix “only” two modes, as well as the choice of which two, is not uniquely prescribed by any physical principle, so we do not expect eq 9 to fit the PO spectra perfectly. Furthermore, using a constant self-energy is at odds with the fully general frequency-dependent expression (eq S29 in the section SXI.2 in the Supporting Information(27,52−55)).

An alternative way to account for the observed PO patterns is by adopting a second-order Raman scattering description,^27^ as this will afford a sum of squares expression similar to eq 9. However, this requires a higher level of perturbation, does not explain the temperature dependence, and is at odds with the observed sharp peaks and high scattering intensities. We therefore argue that the two-mode model is the minimally generalized treatment capable of explaining the temperature evolution of the observed PO patterns, and appreciable contributions from off-diagonal self-energy components are a necessary qualification of any full description of Raman scattering in these systems.

### 3.3 Different Manifestations of Anharmonicity in Light Scattering

**Figure 4:**
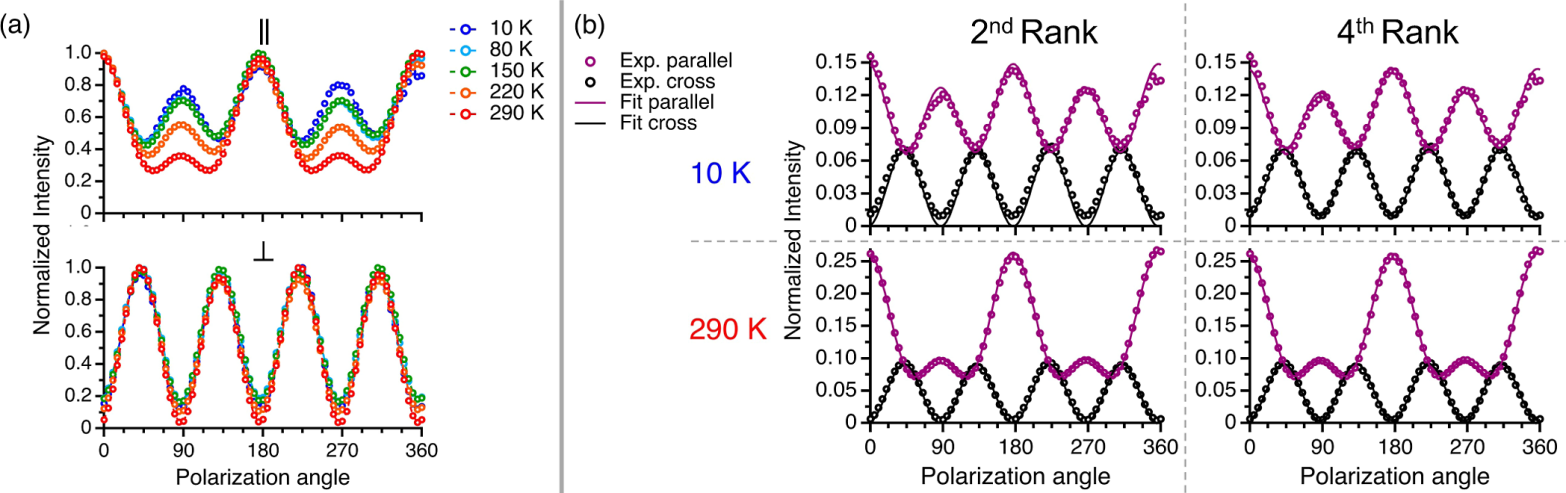
(a) PO Raman response of ω_1_ from 10 to 290 K in parallel (top panel) and cross (bottom panel) configurations. (b) Fit results of the PO Raman response of ω_1_ at 10 K (top panels) and 290 K (bottom panels) using the second-rank Raman tensor formalism (left panels) and the fourth-rank Raman tensor formalism (right panels).

The spectra and fits for another peak, ω_1_, are presented in Figure 4. Unlike the PO patterns of ω_4_, fitting eq 1 to ω_1_ proved satisfactory for all temperatures, with the generalized fourth-rank tensor offering only a modest improvement (Figure 4b). Importantly, the PO pattern of ω_1_ still exhibits a clear evolution with temperature (Figure 4a), which cannot be explained by harmonic theory.

These two different scenarios demonstrate an important distinction in our interpretation of the mode mixing described by the self-energy off-diagonal components. If an irrep-specific second-rank tensor is able to capture the PO pattern, then same-irrep couplings have primarily contributed to the peak. Because the anharmonic spectral function is temperature-dependent, the weight of each contributing mode can change with temperature, altering the PO response. Alternatively, whenever we observe a departure from the fourfold cross pattern described above, a fourth-rank tensor must be invoked, and mixing between different irreps must be the culprit. We can therefore immediately determine the spectral feature ω_4_ (Figure 2) arises from appreciable ⟨A_g_,B_g_⟩ interactions, even without the benefit of an explicit fit. This statement can potentially be tested further by numerical simulations,^26^ or a 2D-Raman measurement,^63^ where inter-mode correlations are directly evaluated.

Note that examination of any single BTBT spectrum would not hint at any extraordinary anharmonic effects, as the spectral features themselves are not particularly broad, nor do they display exceptional softening upon heating. The significance of off-diagonal components is therefore not necessarily correlated with the overall magnitude of the self-energy term. Indeed, perhaps, it was the relative conventional spectrum shape that made the observation possible.

## 4 Symmetry and Light Scattering

The two-mode model of Section 3.2 allowed for a finite correlation between vibrational modes with different irreps. Strictly speaking, these should vanish out of symmetry considerations.^64^ This apparent contradiction is resolved by the fact that Raman scattering does not probe true Γ-point phonons. Momentum conservation in the scattering process confers some finite momentum ***q*** to the vibrational excitation, determined by the measurement geometry and crystal orientation. Both participating Γ-irreps map into identical irreps of *G*(***q***), the group of the wave-vector, so their coupling is allowed.^64^ In other words, the Green’s function remains invariant under the symmetry operations of the ***q***-vector and therefore need not vanish. In this sense, our use of the Γ-point susceptibility derivatives **χ**_Γ_ is an approximation, retrospectively justified by the successful global fit.

Inspection of eqs 4–7 shows that a bottom-up treatment of anharmonic scattering requires a large number of parameters. However, unlike the irrep-specific material property **χ**_λ_, the fourth-rank tensor *I*_μνξρ_ is an observable, which means it must conform to all symmetry constraints of the crystal space group, regardless of the underlying scattering mechanism. In the language of factor group analysis, *I*_μνξρ_ must have Γ_1_ symmetry.^65^ This places significant constraints on its tensor form, so even in the case of the low-symmetry monoclinic space group of BTBT, (*P*2_1_/*c*), the number of fitting parameters in our back-scattering geometry reduces to four.

Our distinction between the underlying dipole radiation mechanism, given in terms of a second-rank tensor, and the actual observable intensity, given in terms of a fourth-rank tensor, is completely general. The symmetry constraints of correlation functions like eq 3 have been described for a variety of scattering schemes.^66^ This conceptual partition between observable and model was previously employed in inelastic light scattering from isotropic media.^67,68^ Considering the symmetry-constrained fourth-rank tensor as the fundamental material property governing light scattering might therefore benefit other spectroscopic scenarios in the solid-state, such as exciton or magnon scattering.

## 5 Conclusions and Outlook

In conclusion, we have demonstrated the unique role of anharmonicity in the polarization dependence of inelastic light scattering at finite temperatures. We showed that the observed PO patterns of BTBT and other organic crystals can only be explained by generalizing the tensor structure governing Raman scattering to the fourth rank. This generalization is theoretically justified by allowing off-diagonal self-energy components to persist, which represents the mixing between vibrational modes and re-normalization of their eigenvectors.

The proposed fourth-rank model was realized by a sum of susceptibility tensor products with different irreducible representations and successfully tested by performing a global fit to the PO response of adjacent spectral features.

It is desirable to predict which vibrational modes will couple as well as understand the temperature evolution of the off-diagonal self-energy components that describe them. Perturbative methods offer a recipe for their calculation, given a known potential surface,^53,56,69^ but mapping chemical structure to such a surface is a formidable task in and of itself. Generally, the effect is expected to depend on the whole of the vibrational dispersion relation and require methods that provide the effective temperature-dependent potential surface,^27,70^ to fully understand.

## Supporting Information Available

Raman measurement details; powder X-ray diffraction measurements of BTBT; raw data for temperature and polarization-dependent Raman spectra; PO benchmark measurements for Silicon and chloroform; details of deconvolution and all fitting procedures; full temperature evolution of BTBT PO response; discussion of positive semi-definite fourth-rank tensors; raw data, analysis, and discussion of the effect in anthracene and pentacene; and theoretical review of inelastic light scattering from crystals at finite temperatures using Green’s function formulation (PDF)
