# Supplementary material for: Phonon–Phonon Interactions in the Polarization Dependence of Raman Scattering
Source: J Phys Chem C Nanomater Interfaces. 2023 Aug 31;127(36):18099–106. doi: 10.1021/acs.jpcc.3c03850 (PMC10510386; doi:10.1021/acs.jpcc.3c03850)
Supplement: Supplementary file 1 — jp3c03850_si_001.pdf [file jp3c03850_si_001.pdf]

Supporting Information

Phonon-Phonon Interactions in the Polarizarion Dependence of Raman Scattering

Nimrod Benshalom,[1,†] Maor Asher,[1,†] Rémy Jouclas,[2] Roman Korobko,[1] Guillaume Schweicher,[2] Jie Liu[2], Yves Geerts,[2,3] Olle Hellman,[4,5,*] and Omer Yaffe[1,*]

[1]*Department of Chemical and Biological Physics, Weizmann Institute of Science, Rehovot 76100, Israel*

[2]*Laboratoire de Chimie des Polymères, Universite Libre de Bruxelles (ULB), 1050 Brussels, Belgium*

[3]*International Solvay Institutes for Physics and Chemistry, 1050 Brussels, Belgium*

[4]*Department of Physics, Chemistry and Biology (IFM), Linköping University, SE-581 83, Linköping, Sweden*

[5]*Department of Molecular Chemistry and Material Science, Weizmann Institute of Science, Rehovot 76100, Israel*

† These authors contributed equally.

∗ olle.hellman@liu.se

∗ omer.yaffe@weizmann.ac.il

SI Measurement details

Crystals Growth and characterization: [1]benzothieno[3,2-*b*]benzothiophene (BTBT) single-crystals were grown by leaving a saturated BTBT solution in chloroform in open air at room temperature until complete chloroform evaporation. We confirmed the crystal structure and high phase purity of the crystals by performing XRD measurements (see Sec. SII).

Temperature-Dependent PO Raman: A custom-built Raman system was used to conduct the Raman measurements. The system included a 785 nm Toptica diode laser with intensity of around 30 mW on the sample. To control the polarization of the incident and scattered light for the polarization-dependent measurements (5° steps), rotating half-wave plates and a polarizer-analyzer combination were used. The system included a 50x objective. Notch filters are included in the system to allow access to the low-frequency region (>10 cm^{-1}) and simultaneous acquisition of the Stokes and anti-Stokes signal. The system is based on a 1 m long Horiba FHR-1000 dispersive spectrometer with a 1800 mm^{-1} grating. The spectral resolution was approximately 0.15 cm^{-1}. The temperature was set and controlled by a Janis cryostat ST-500 and a temperature controller by Lakeshore model 335.

SII Powder X-ray diffraction measurements of BTBT

Preferred orientation and phase purity of BTBT were analyzed using powder X-ray diffraction. The experiment was performed at room temperature. The Compound was finely ground using a mortar and pestle for phase confirmation measurement. Single crystals were mounted over the sample holder for preferential orientation analysis. For BTBT the measurements were conducted on a Panalytical Empyrean diffractometer using Cu–Kα radiation (λ =1.54178 Å). The diffractometer was set up with reflection-transmission spinner 3.0 configuration, and patterns were collected with 2θ range between 5.0 and 30.0°, steps of 0.1°, time per step of 2.5 s, and rotation of 1 r/s. A calculated pattern was obtained from known crystal structure of BTBT[1;2;3] using Powder Pattern tool on Mercury software.[4;5] The crystalline phase of BTBT was confirmed to have the same phase of the known crystal structures with CSD Refcode PODKEA02[3] (BTBT) as it can be observed in Figure S1 (experimental diffraction pattern in red and calculated diffraction patterns in black). The results show single crystals of BTBT with highly (100) preferred orientation crystallized along [001] direction.

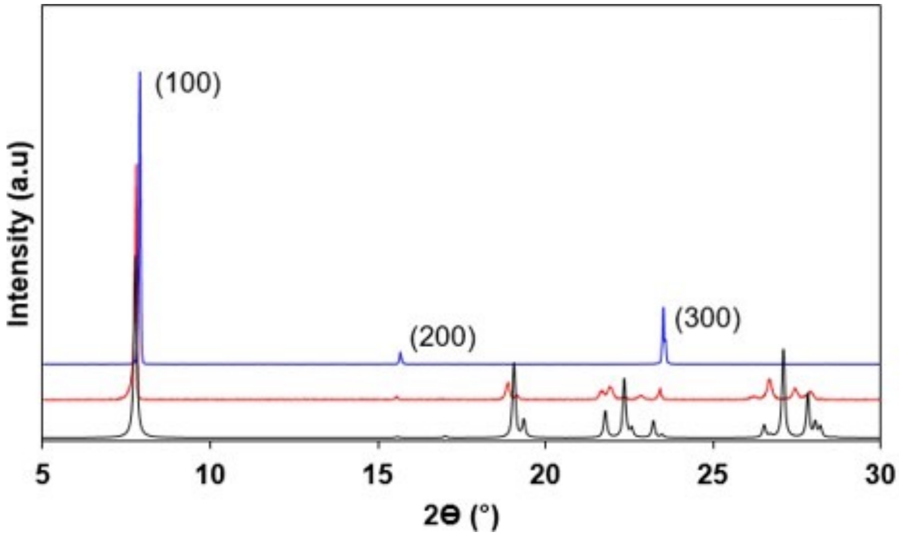

Figure S1: X-Ray diffraction patterns calculated from the known crystal structures (in black), obtained experimentally from powders (in red) and obtained experimentally from single crystals (in blue) of BTBT.

SIII Deconvolution of Raman spectra

To extract the integrated intensity of a spectral feature, we fit the measured Stokes-shift Raman spectra with the product of the Bose-Einstein distribution and a multi-damped Lorentz oscillator line shape,

$$I_{Raman}(\Omega) = \left(\frac{1}{e^{\frac{\hbar\Omega}{k_B T}} - 1} + 1\right) \sum_i \frac{c_i |\Omega| \Gamma_i^3}{\Omega^2 \Gamma_i^2 + (\Omega^2 - \omega_i^2)^2} \tag{S1}$$

Where ω_i, c_i and Γ_i are the position, intensity, and width of each peak, respectively. Ω is the measured frequency (Raman shift), T is the temperature, $\hbar$ is the Planck constant and k_B is the Boltzmann constant. The Lorentz in Equation (S1) is a variation of the Lorentz oscillator, where c is the maximum value of the peak. Given the fitted Lorentzian parameters, the integrated intensity of the spectral feature is

$$I_{int} = [n_{\mathrm{BE}}(\omega_i, T) + 1] \frac{2c_i |\omega_i| \Gamma_i^2}{\sqrt{4\omega_i^2 - \Gamma_i^2}} \left[\frac{\pi}{2} - \tan^{-1}\left(\frac{\Gamma_i^2 - 2\omega_i^2}{\Gamma_i \sqrt{4\omega_i^2 - \Gamma_i^2}}\right)\right], \tag{S2}$$

with n_{BE} standing for an approximated Bose-Einstein constant pre-factor.

SIV The temperature independent polarization-orientation Raman of silicon

Figure S2 presents the PO Raman measurements of a (100) silicon wafer at 10 K and 300 K with the analysis of the polarization dependence of the integrated intensity of the prominent Raman peak (the TO phonon at around 520 cm^{-1}). We performed the measurements and analysis similar to the organic crystals discussed in the main text using the same optical setup. We present these results as an example for an inorganic material that shows a temperature independent PO response, in agreement with the harmonic picture. Furthermore, according to theory, the intensity of the prominent Raman peak should go down to zero at the minimum point.[6] In our measurement, the peak intensity drops to about 1% of its maximum intensity, showing our system suffers very little leakage.

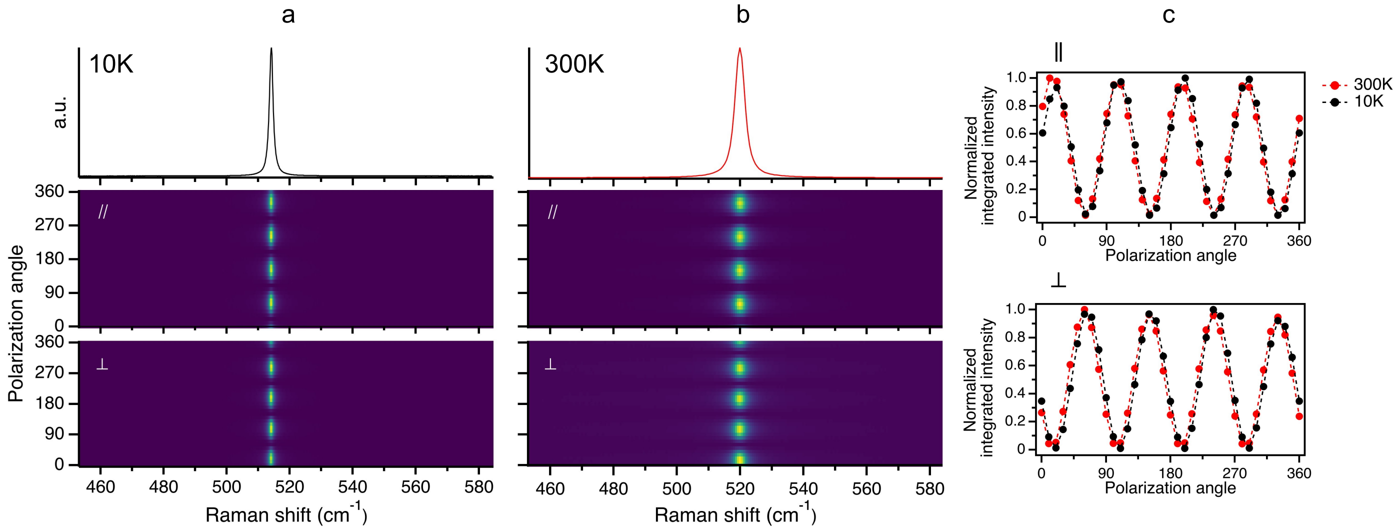

Figure S2: Temperature dependence of the PO Raman of silicon. (a) and (b) presents the PO Raman measurement of silicon at 10 K and 300 K, respectively. The middle and bottom panels present the measurements in parallel and cross configurations, respectively. The top panel presents the unpolarized spectrum (sum over all angles). (c) presents the polarization dependence of the integrated intensity of the prominent Raman peak. The top and bottom panels present the results for parallel and cross configurations, respectively.

SV Polarization-orientation Raman of chloroform

Figure S3 presents the PO Raman measurement of liquid chloroform at room temperature. The measurement was performed similarly to the organic crystals discussed in the main text using the same optical setup. Since there is no long-range order in a liquid, we expect the measurement to be polarization independent. This is why measuring the PO response of a liquid is helpful as a test for the system response to the change in polarization angle. Our results show that the PO response of the vibrations of chloroform is completely polarization-independent - proving our systems response is minimal.

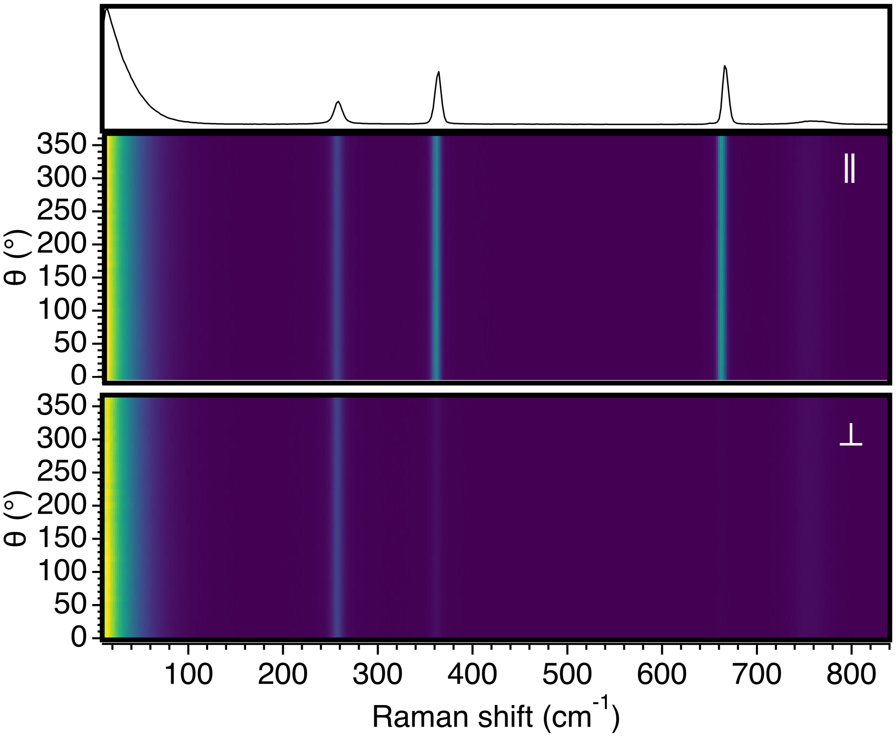

Figure S3: Polarization dependent measurement of liquid chloroform in parallel and cross configurations. The top panel shows a typical spectrum in the parallel configuration.

SVI Temperature dependent low-frequency Raman spectroscopy of BTBT

Figure S4 shows the temperature dependent low-frequency Raman spectroscopy spectra of BTBT, from 10 K to 290 K at 10 K increments. We see only a gradual redshift and broadening of the spectrum as temperature increases. No abrupt changes to the Raman spectrum indicate there is no phase transition in the measured temperature range.

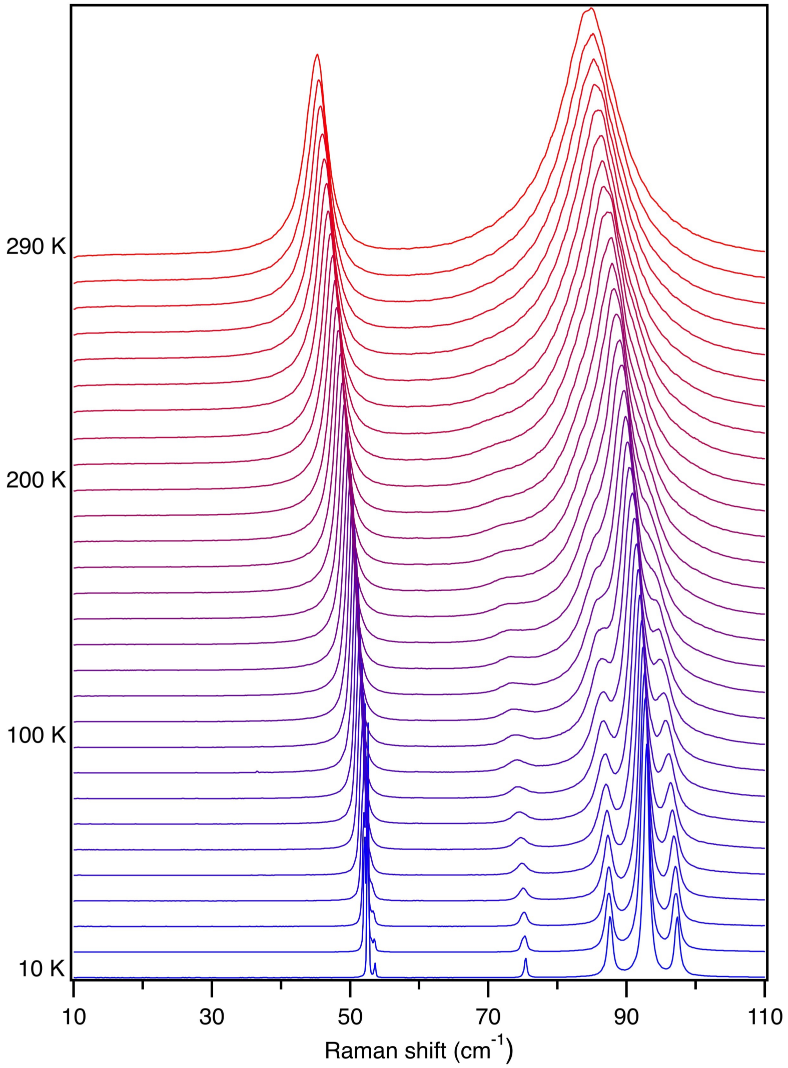

Figure S4: Temperature dependent low-frequency Raman of BTBT. The spectra were normalized and shifted up for clarity. The temperature increment is 10 K.

Figure S5 shows the temperature dependence of the peaks position and full-width-half-maximum (FWHM) of the lattice vibrations, extracted from fitting the spectra according to Sec. SIII. The trend with temperature of vibrational frequency and FWHM is primarily linear.

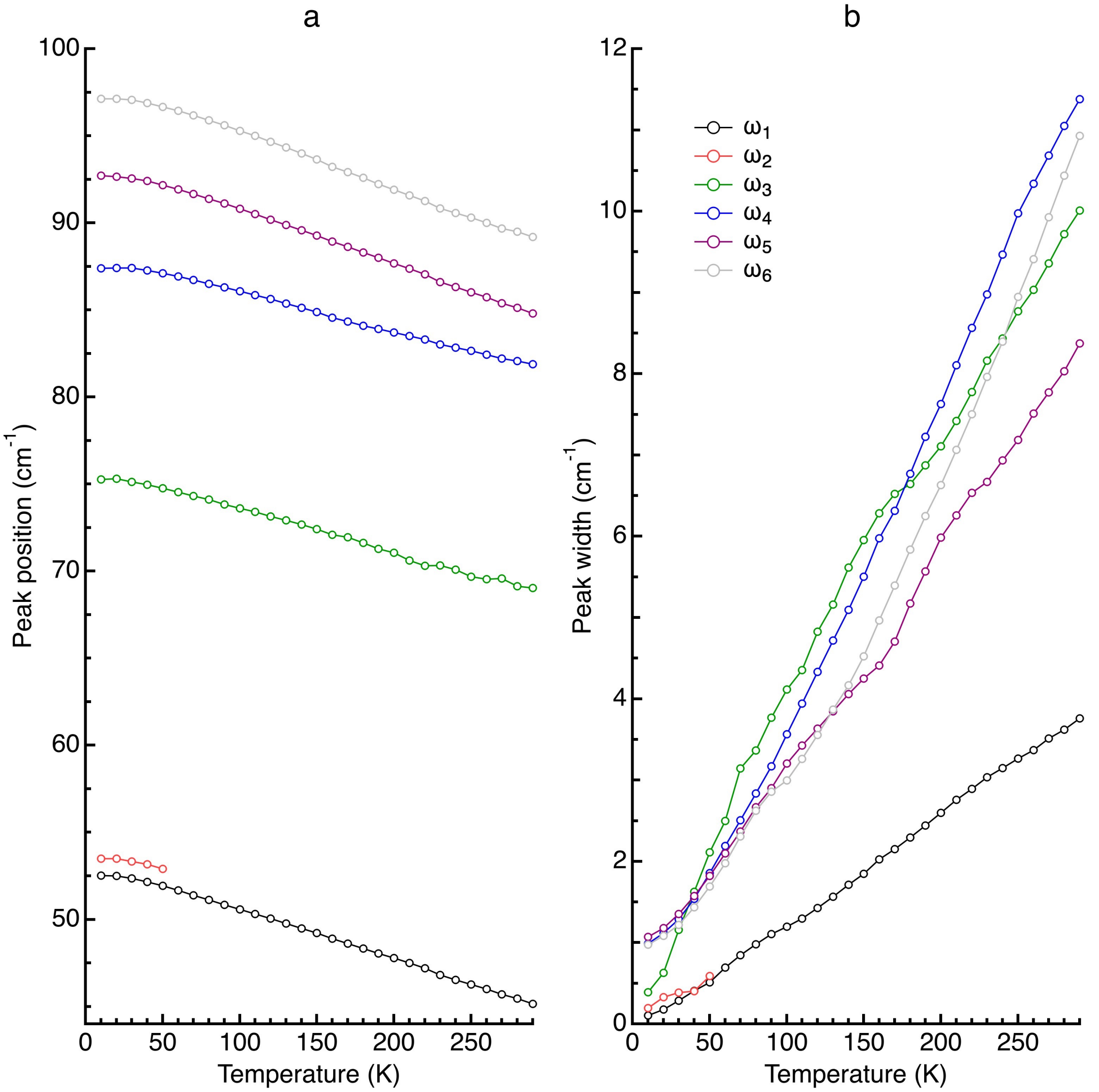

Figure S5: The temperature dependence of the vibrational frequencies (a) and FWHM (b) of the lattice vibrations of BTBT.

SVII Raman polarization-orientation color maps of BTBT

Figure S6 presents the raw data obtained from the PO Raman measurements for both parallel and cross configurations for a single crystal of BTBT at 10, 80, 150, 200, and 290 K.

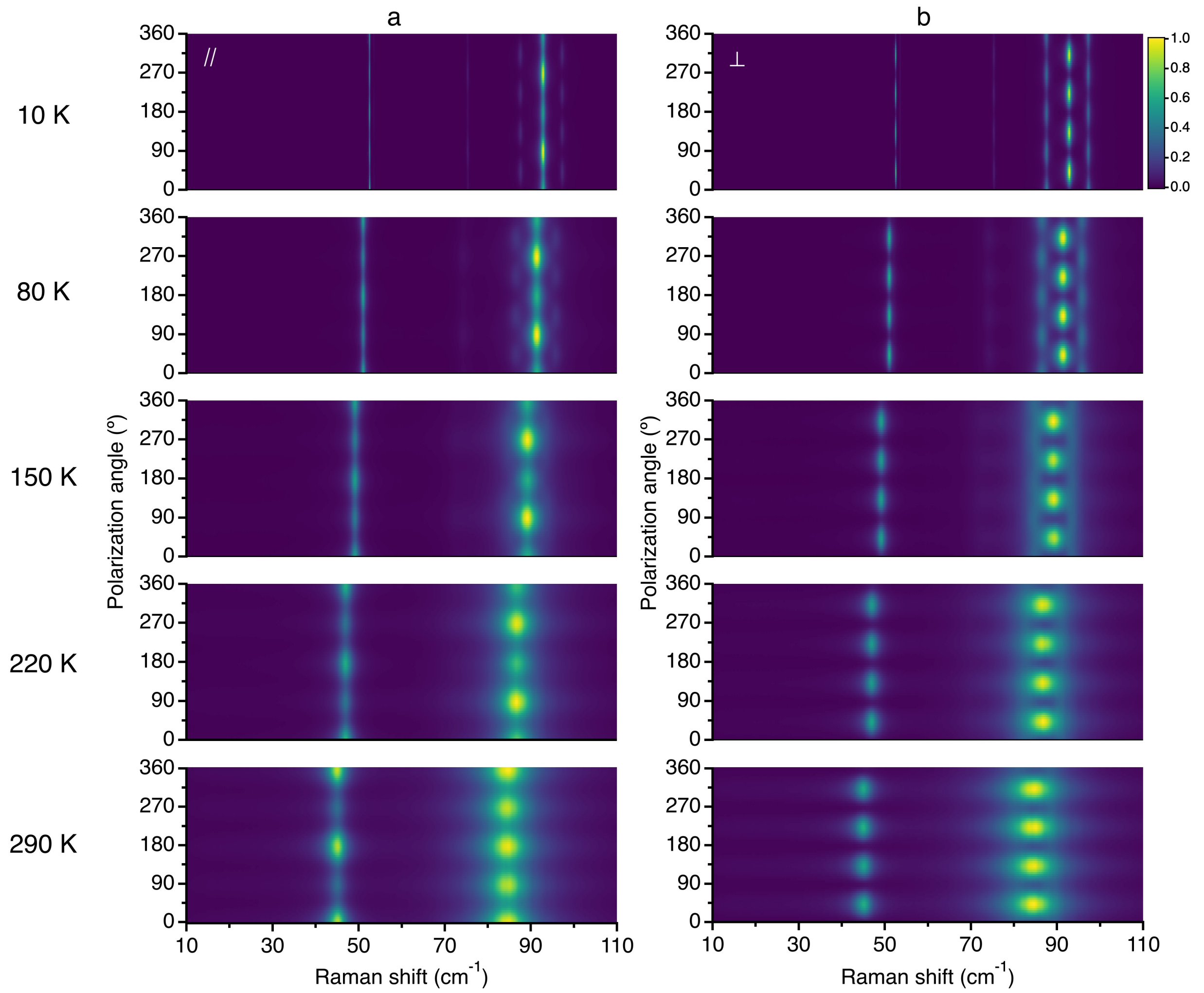

Figure S6: Raw PO Raman of BTBT in (a) parallel and (b) cross configurations at 10 K, 80 K, 150 K, 220 K and 290 K.

SVIII Temperature evolution in the Raman polarization-orientation response of BTBT

Figure S7 presents the temperature evolution of the PO pattern of each separate peak in the Raman spectrum of BTBT. The results show the PO Raman response of all BTBT lattice vibrations evolves with temperature.

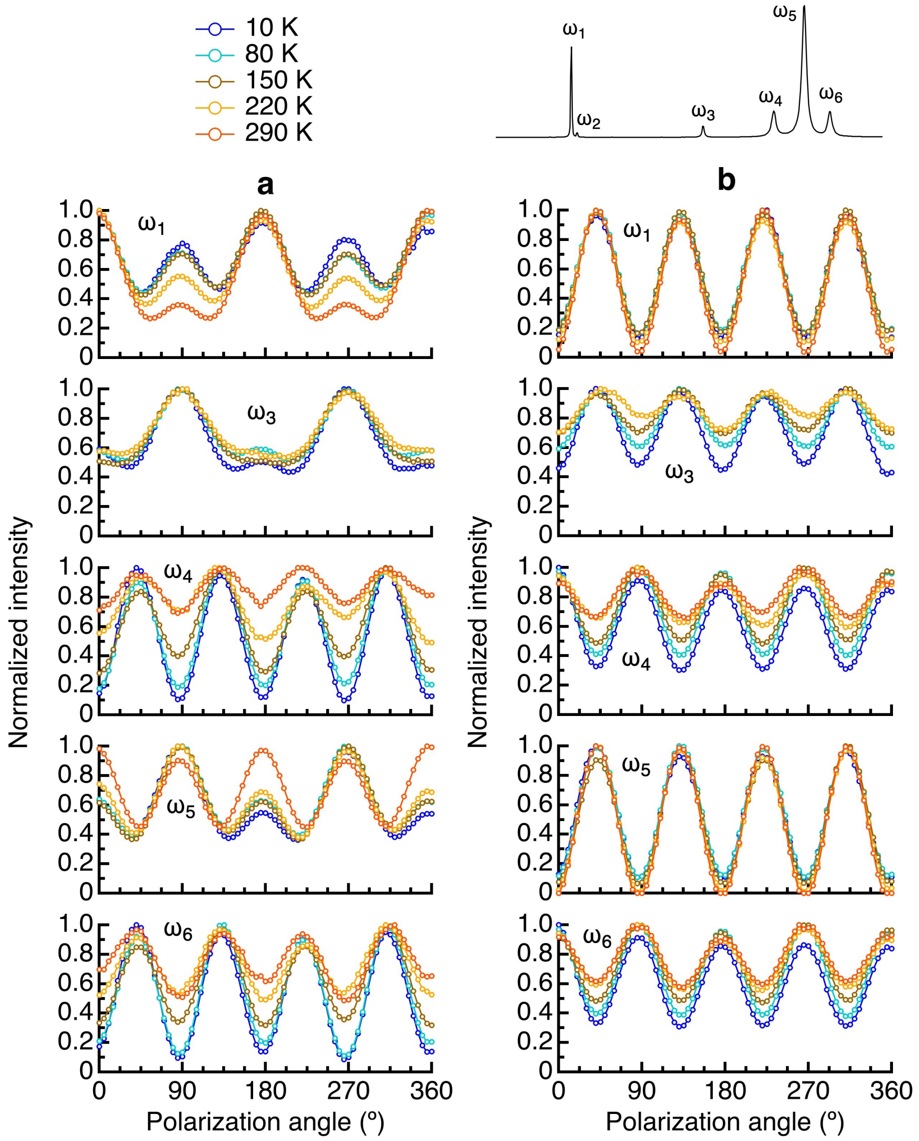

Figure S7: The temperature evolution of the PO dependence for the low-frequency peaks of BTBT in (**a**) parallel and (**b**) cross configurations. The intensities were normalized for each mode at each temperature.

SIX Fitting the PO response of BTBT

SIX.1 Data preparation and factor group analysis for BTBT

Section SVII presents the contour plots of the PO Raman measurement of BTBT at 10, 80, 150 220, and 290 K in parallel and cross configurations. The measurements were performed as described in Sec. SI. To extract the polarization dependence of the integrated intensity of each peak we fit the spectra at each polarization angle to a multi-damped Lorentz oscillator (see section SIII). The dotted lines in Figures S8 and S9 show the integrated intensity of each damped Lorentz oscillator with respect to the excitation polarization angle. Next, for the second-rank Raman tensor formalism we extract the form of the Raman tensors, as well as the expected number of lattice modes and their vibrational symmetry, by using factor group analysis with the relevant space group (monoclinic, $P2_1/c$).[7] For BTBT, factor group analysis predicts 6 Raman-active lattice libration modes, and indeed our measured spectra showed exactly six distinguishable Raman peaks.

SIX.2 Fitting the PO with a second-rank tensor

The symmetry allowed Raman tensors for $3A_g$ and $3B_g$ are:[8]

$$\mathcal{R}_{A_g} = \begin{pmatrix} a & 0 & e \\ 0 & b & 0 \\ e & 0 & c \end{pmatrix}, \qquad \mathcal{R}_{B_g} = \begin{pmatrix} 0 & d & 0 \\ d & 0 & f \\ 0 & f & 0 \end{pmatrix}. \tag{S3}$$

We perform a global fit to the integrated intensity in both parallel and cross configurations using the second-rank Raman tensor formalism described by Eq. (1) in the main text (see Ref.[9] for more details regarding the fitting process).

Figure S8 shows the results for this fitting procedure at all measured temperatures. The 10 K fits show that ω_1, ω_3, and ω_5 are A_g modes, while ω_4 and ω_6 are B_g modes. The intensity of ω_2 was too weak to extract its polarization dependence reliably. The fit gets worse for ω_3, ω_4 and ω_6 as temperature increases, while for ω_1 and ω_5 the fit is successful in all temperatures.

To further challenge the necessity of a fourth-rank tensor, we attempted fitting a symmetry-

relaxed second-rank tensor:

$$\tilde{\mathcal{R}} = \begin{pmatrix} a & d_1 & e_1 \\ d_2 & be^{i\delta} & f_1 \\ e_2 & f_2 & c \end{pmatrix}, \tag{S4}$$

where we introduce a relative phase δ to account for possible birefringence effects,[10] and allow asymmetric components (effectively relaxing time-symmetry).[11] Adapted to our back-scattering geometry, $\tilde{\mathcal{R}}$ has five independent parameters (a, b, d_1, d_2, δ). As Fig. S8 demonstrates, this generalized second-rank tensor form was still unable to fit the observed PO pattern of ω_4.

SIX.3 Fitting the PO with a fourth-rank tensor

Applying the symmetry constraints of the relevant space group ($P2_1/c$) along with the intrinsic left and right minor symmetries, and retaining only the components relevant for our back-scattering geometry, the effective fourth-rank tensor used for fitting the PO patterns was:

$$I_{\mu\nu\xi\rho} = \begin{pmatrix} \begin{pmatrix} a & 0 & 0 \\ 0 & b & 0 \\ 0 & 0 & 0 \end{pmatrix} & \begin{pmatrix} 0 & d & 0 \\ d & 0 & 0 \\ 0 & 0 & 0 \end{pmatrix} & \begin{pmatrix} 0 & 0 & 0 \\ 0 & 0 & 0 \\ 0 & 0 & 0 \end{pmatrix} \\ \begin{pmatrix} 0 & d & 0 \\ d & 0 & 0 \\ 0 & 0 & 0 \end{pmatrix} & \begin{pmatrix} b & 0 & 0 \\ 0 & c & 0 \\ 0 & 0 & 0 \end{pmatrix} & \begin{pmatrix} 0 & 0 & 0 \\ 0 & 0 & 0 \\ 0 & 0 & 0 \end{pmatrix} \\ \begin{pmatrix} 0 & 0 & 0 \\ 0 & 0 & 0 \\ 0 & 0 & 0 \end{pmatrix} & \begin{pmatrix} 0 & 0 & 0 \\ 0 & 0 & 0 \\ 0 & 0 & 0 \end{pmatrix} & \begin{pmatrix} 0 & 0 & 0 \\ 0 & 0 & 0 \\ 0 & 0 & 0 \end{pmatrix} \end{pmatrix} \tag{S5}$$

Figure S9 shows the fit results for the same PO Raman data set by using the fourth-rank Raman tensor formalism. Here we see that the fit is excellent at all temperatures. These results are discussed in the main text.

SIX.4 Global fit with the two-mode model

The full PO data for spectral features ω_4 and ω_5 at 10 K was globally fitted using the two-mode model discussed in the main text, with birefringence effects incorporated as in the harmonic case of Sec. S9.2, introducing the additional parameter δ.

In practice, this means *simultaneously* fitting the spectral range 85-95 cm^{-1} for all 144 spectra (72 polarization angles for each polarization configuration) to:

$$\sigma(\Omega) \propto \sum_{\mu\nu\xi\rho} n_\mu n_\xi I_{\mu\nu\xi\rho}(\Omega) E_\nu E_\rho, \tag{S6}$$

with

$$I_{\mu\nu\xi\rho}(\Omega) = [n_{BE}(\Omega, T) + 1] \left[\chi^{*\mu\nu}_{\mathrm{A_g}} \chi^{\xi\rho}_{\mathrm{A_g}} J_{\mathrm{A_g A_g}} + \chi^{*\mu\nu}_{\mathrm{B_g}} \chi^{\xi\rho}_{\mathrm{B_g}} J_{\mathrm{B_g B_g}} \right], \tag{S7}$$

and

$$\chi_{\mathrm{A_g}} = \begin{pmatrix} a & 0 & e \\ 0 & be^{i\delta} & 0 \\ e & 0 & c \end{pmatrix}, \qquad \chi_{\mathrm{B_g}} = \begin{pmatrix} 0 & de^{i\delta} & 0 \\ de^{i\delta} & 0 & fe^{i\delta} \\ 0 & fe^{i\delta} & 0 \end{pmatrix}. \tag{S8}$$

Applying our back-scattering geometry to Eq. (S6) gives the following final fitted expressions for parallel ($\|$) and cross ($\perp$) configurations:

$$\sigma_\| \propto J_{\mathrm{AgAg}} a^2 \cos^4(\Delta\theta) + 2J_{\mathrm{AgAg}} ab \cos(2\delta) \cos^2(\Delta\theta) \sin^2(\Delta\theta)^2 + \\ 4J_{\mathrm{BgBg}} d^2 \cos^2(\Delta\theta) \sin^2(\Delta\theta) + J_{\mathrm{AgAg}} b^2 \sin^4(\Delta\theta), \tag{S9}$$

$$\sigma_\perp \propto J_{\mathrm{AgAg}} a^2 \cos^2(\Delta\theta) \sin^2(\Delta\theta)^2 + J_{\mathrm{AgAg}} b^2 \cos^2(\Delta\theta) \sin^2(\Delta\theta)^2 - \\ 2J_{\mathrm{BgBg}} d^2 \cos^2(\Delta\theta) \sin^2(\Delta\theta) - 2J_{\mathrm{AgAg}} ab \cos(2\delta) \cos^2(\Delta\theta) \sin^2(\Delta\theta)^2 + \\ J_{\mathrm{BgBg}} d^2 \cos^4(\Delta\theta) + J_{\mathrm{BgBg}} d^2 \sin^4(\Delta\theta), \tag{S10}$$

where $\Delta\theta = \theta - \theta_0$ is determined by the incident polarization θ, controlled in experiment, and θ_0 is a global angle determined by the crystal orientation. The spectral frequency dependency is contained within the spectral functions $J_{\lambda\lambda'}(\Omega)$ and the $[n_{BE}(\Omega, T) + 1]$ factor.

The full derivation of $J_{\lambda\lambda'}$ for the two-mode model is given in the main text, equations (6), (7), (9). This yielded the final function used in the global fit (using the shorthand $\mathrm{A_g} \equiv 1$ and $\mathrm{B_g} \equiv 2$):

$$J_{11}(\Omega) = \frac{\Gamma_2\left(-\gamma^*\gamma - \Gamma_1\Gamma_2 + \left(\frac{\Omega^2-\omega_1^2}{2\omega_1}\right)\left(\frac{\Omega^2-\omega_2^2}{2\omega_2}\right)\right) - \left(\frac{\Omega^2-\omega_2^2}{2\omega_2}\right)\left(\Gamma_1\left(\frac{\Omega^2-\omega_2^2}{2\omega_2}\right) + \Gamma_2\left(\frac{\Omega^2-\omega_1^2}{2\omega_1}\right)\right)}{\left(-\gamma^*\gamma - \Gamma_1\Gamma_2 + \left(\frac{\Omega^2-\omega_1^2}{2\omega_1}\right)\left(\frac{\Omega^2-\omega_2^2}{2\omega_2}\right)\right)^2 + \left(\Gamma_1\left(\frac{\Omega^2-\omega_2^2}{2\omega_2}\right) + \Gamma_2\left(\frac{\Omega^2-\omega_1^2}{2\omega_1}\right)\right)^2}, \quad \text{(S11)}$$

with $J_{\mathrm{B_gB_g}}$ being the same only with swapped irrep indices ($\mathrm{A_g} \leftrightarrow \mathrm{B_g}$).

The full list of fit parameters is therefore:

1. ω_i - the peak resonance
2. Γ_i - the peak width
3. θ_0 - global incident polarization off-set

The three parameters mentioned above are identical to those used to extract the integrated intensity in the harmonic analysis. They govern the peak shape, but not the PO pattern. The parameters that create the PO response in our two-mode model are:

4. a - susceptibility derivative for $\mathrm{A_g}$ normal mode
5. b - susceptibility derivative for $\mathrm{A_g}$ normal mode
6. d - susceptibility derivative for $\mathrm{B_g}$ normal mode
7. δ - phase retardance due to birefringence
8. γ - off-diagonal self-energy component

which gives a total of five parameters for two spectral peaks, *the same* as in the generalized second-rank formalism that failed to account for the PO response of the *single* ω_4 peak.

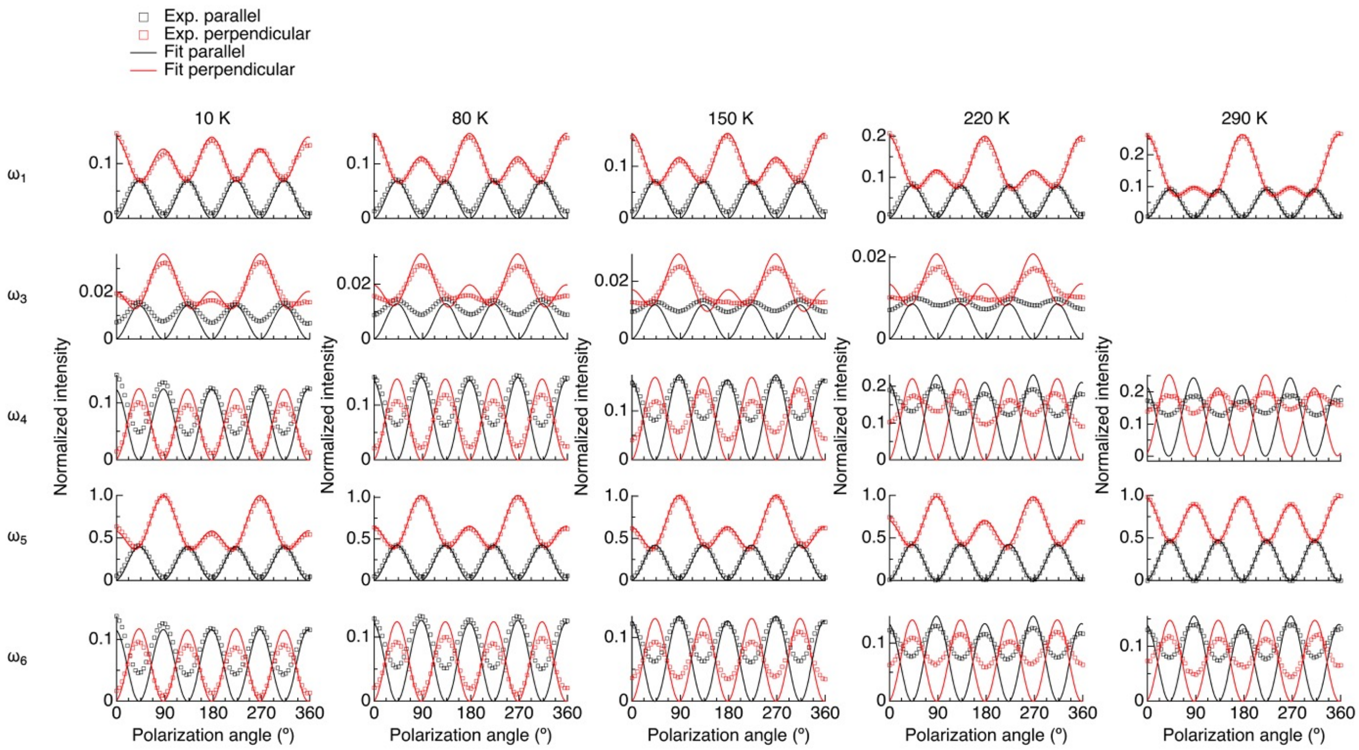

Figure S8: Fitting results for the PO Raman response of BTBT at different temperatures, using a general second-rank Raman tensor.

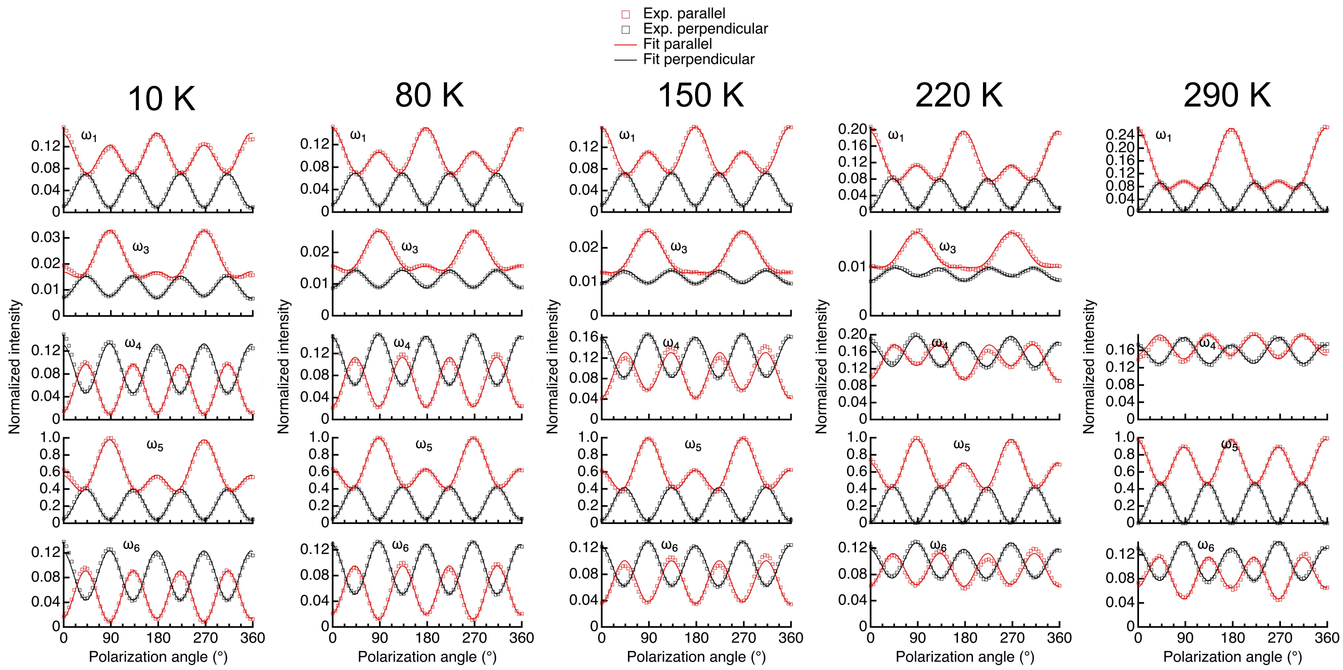

Figure S9: Fitting results for the PO Raman response of BTBT at different temperatures, showing the successful fit obtained by using the fourth-rank formalism.

SIX.5 Positive semi-definite fourth-rank tensors

Unlike the harmonic expression (Eq. (1) in main text), using the general fourth-rank tensor of Eq. (S6) can yield non-positive values. This poses no problem when used for fitting experimental data, but since scattering cross-sections can not be negative, this technically places further constraints on the general tensor form. Because our fourth-rank tensor has the same symmetry of the elastic stiffness tensor, we can apply the positive semi-definiteness constraint by adopting Voigt notation, where the tensor is re-written as a 6X6 matrix.[12;13]

We begin by defining the extended vectors $\tilde{\boldsymbol{n}}$ and $\tilde{\boldsymbol{E}}$:

$$\tilde{\boldsymbol{n}} = \begin{pmatrix} n_x^2 \\ n_y^2 \\ n_z^2 \\ n_y n_z \\ n_x n_z \\ n_x n_y \end{pmatrix}; \qquad \tilde{\boldsymbol{E}} = \begin{pmatrix} E_x^2 \\ E_y^2 \\ E_z^2 \\ E_y E_z \\ E_x E_z \\ E_x E_y \end{pmatrix}. \tag{S12}$$

If we write the following 6X6 matrix in terms of the fourth-rank tensor terms:

$$\tilde{I} = \begin{pmatrix} I_{1111} & I_{1212} & I_{1313} & 2I_{1213} & 2I_{1113} & 2I_{1112} \\ I_{1212} & I_{2222} & I_{2323} & 2I_{2223} & 2I_{1223} & 2I_{1222} \\ I_{1313} & I_{2323} & I_{3333} & 2I_{2333} & 2I_{3313} & 2I_{1323} \\ 2I_{1213} & 2I_{2223} & 2I_{2333} & 2\left(I_{2233}+I_{2323}\right) & 2\left(I_{1233}+I_{1323}\right) & 2\left(I_{1223}+I_{1322}\right) \\ 2I_{1113} & 2I_{1223} & 2I_{1333} & 2\left(I_{1233}+I_{1323}\right) & 2\left(I_{1133}+I_{1313}\right) & 2\left(I_{1123}+I_{1213}\right) \\ 2I_{1112} & 2I_{1222} & 2I_{1323} & 2\left(I_{1223}+I_{1322}\right) & 2\left(I_{1123}+I_{1213}\right) & 2\left(I_{1122}+I_{1212}\right) \end{pmatrix}, \tag{S13}$$

then the expression

$$\tilde{I} = \tilde{\boldsymbol{n}} \cdot \tilde{I} \cdot \tilde{\boldsymbol{E}} = \sum_{\alpha\beta} \tilde{I}_{\alpha\beta} n_\alpha E_\beta \tag{S14}$$

is equivalent to the cross-section defined by Eq. (S6).

It is now straightforward to guarantee the positive semi-definiteness of Eq. (S14) by demanding all eigenvalues of the symmetric matrix $\tilde{I}$ to be non-negative.[14] This will generally yield intractable

expressions. Even after applying the spatial symmetry constraints for BTBT (C_{2h} point group) the scattering tensor still has 14 independent variables with the Voigt-like matrix:

$$\tilde{I}_{C_{2h}} = \begin{pmatrix} I_{1111} & I_{1212} & I_{1313} & 0 & 2I_{1113} & 0 \\ I_{1212} & I_{2222} & I_{2323} & 0 & 2I_{1223} & 0 \\ I_{1313} & I_{2323} & I_{3333} & 0 & 2I_{1333} & 0 \\ 0 & 0 & 0 & 2\left(I_{2233}+I_{2323}\right) & 0 & 2\left(I_{1223}+I_{1322}\right) \\ 2I_{1113} & 2I_{1223} & 2I_{1333} & 0 & 2\left(I_{1133}+I_{1313}\right) & 0 \\ 0 & 0 & 0 & 2\left(I_{1223}+I_{1322}\right) & 0 & 2\left(I_{1122}+I_{1212}\right) \end{pmatrix} \tag{S15}$$

which has complicated eigenvalues.

In the high-symmetry case of the O_h point group, however, the scattering tensor has only three independent variables and the Voigt-like matrix is

$$\tilde{I}_{O_h} = \begin{pmatrix} a & b & b & 0 & 0 & 0 \\ b & a & b & 0 & 0 & 0 \\ b & b & a & 0 & 0 & 0 \\ 0 & 0 & 0 & 2\left(b+c\right) & 0 & 0 \\ 0 & 0 & 0 & 0 & 2\left(b+c\right) & 0 \\ 0 & 0 & 0 & 0 & 0 & 2\left(b+c\right) \end{pmatrix}, \tag{S16}$$

which has only three different eigenvalues, such that the requirement for positive semi-definiteness becomes

$$a-b \geq 0; \qquad b+c \geq 0; \qquad a+2b \geq 0. \tag{S17}$$

SX Raman polarization-orientation measurements of anthracene and pentacene

The full PO Raman spectra of anthracene and pentacene crystals were measured in a similar fashion to that described in Sec. SI (see full measurement and irrep assignment details in[9]). The PO Raman spectra at 10 K for both anthracene and pentacene are presented in Fig. S10.

A procedure similar to that used in BTBT (Sec. SIII) was applied to extract the integrated intensity of each peak, and fit the PO pattern to both a second (Sec. SIX.2) and fourth-rank (Sec. SIX.3) formalism (all three crystals share the same C_{2h} point-group and tensor forms). The results are presented in figures S11 (second rank), S12 (fourth-rank) for anthracene, and figures S13 (second-rank), S14 (fourth-rank) for pentacene. In both cases the second-rank formalism fails to capture the PO of all peaks (specifically, ω_2, ω_6 for anthracene, and ω_6, ω_6, ω_7 for pentacene), whereas the fourth-rank formalism consistently yields a perfect fit.

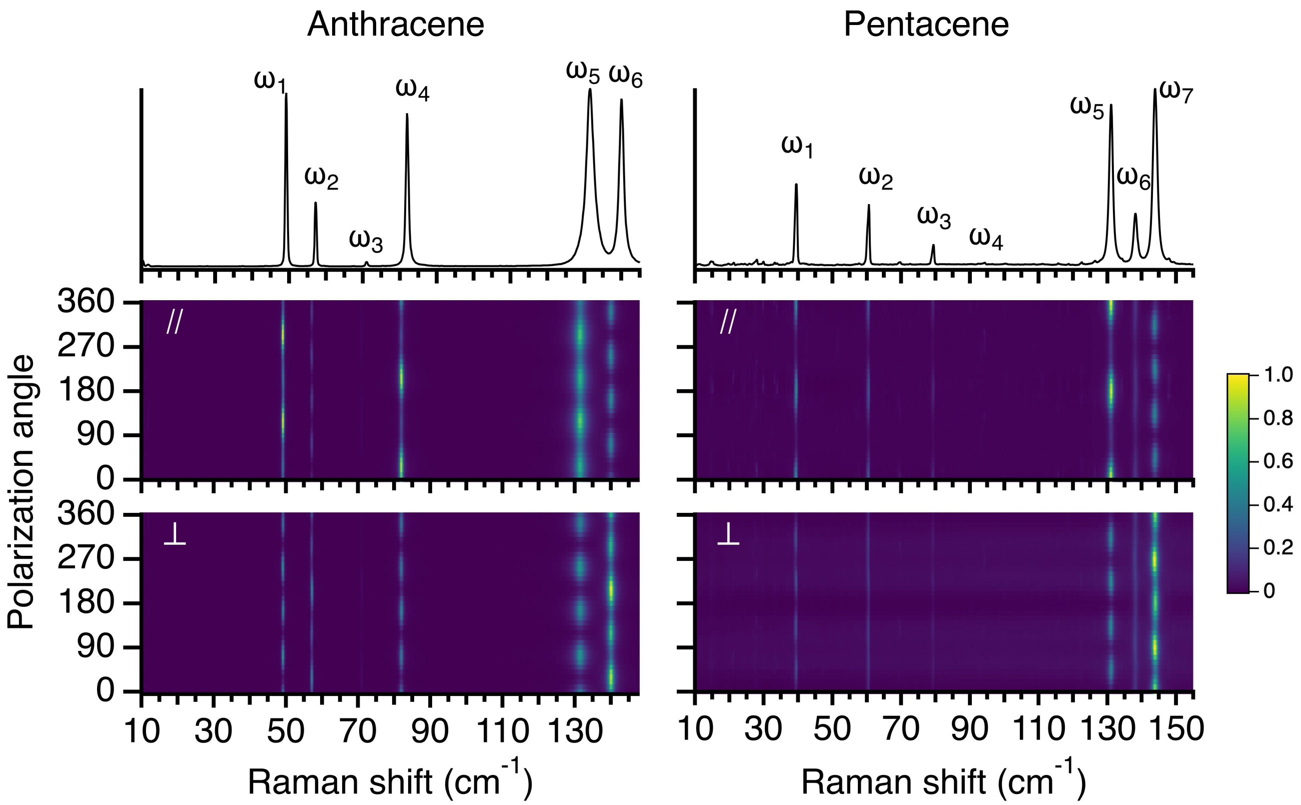

Figure S10: The PO Raman of anthracene (left) and pentacene (right) at 10 K. Top row shows the unpolarized spectra. Middle and bottom rows present the PO for parallel and cross configurations, respectively.

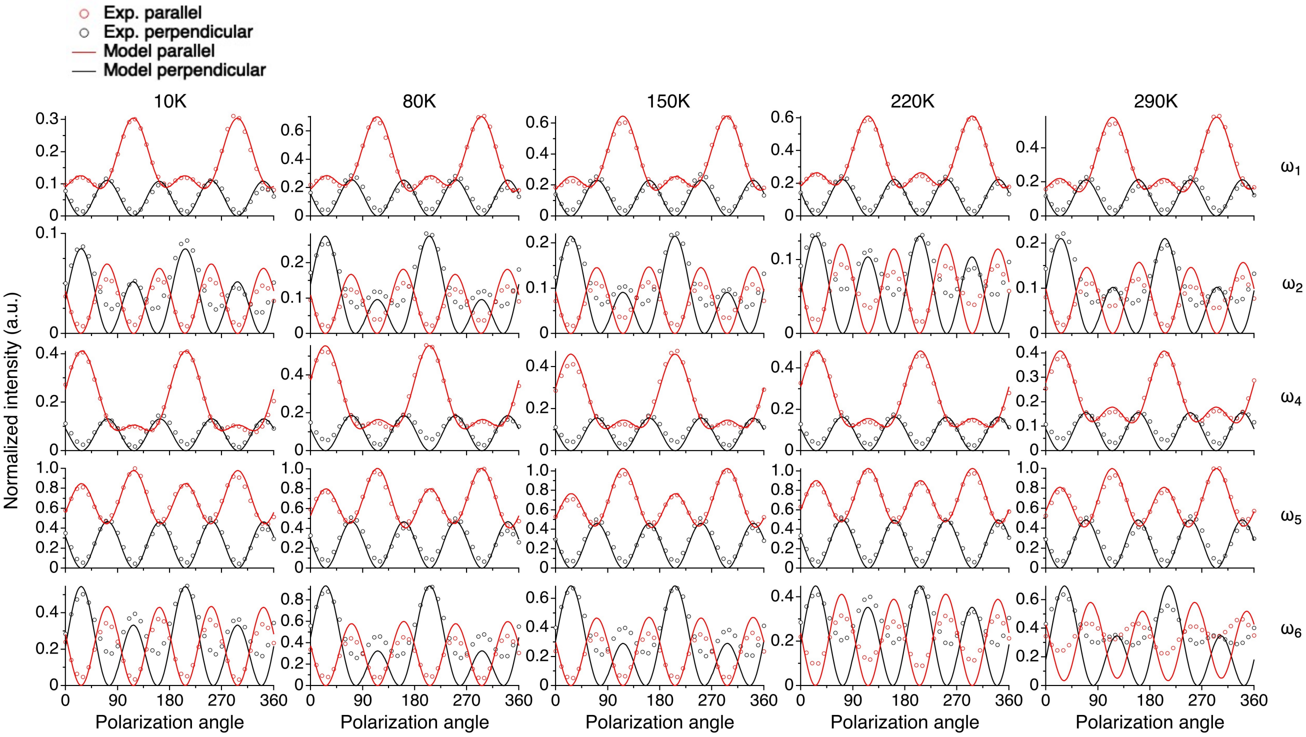

Figure S11: Fitting results for the PO Raman response of anthracene at different temperatures, using a general second-rank Raman tensor. The fit for peaks ω_2, ω_6 is unsatisfactory.

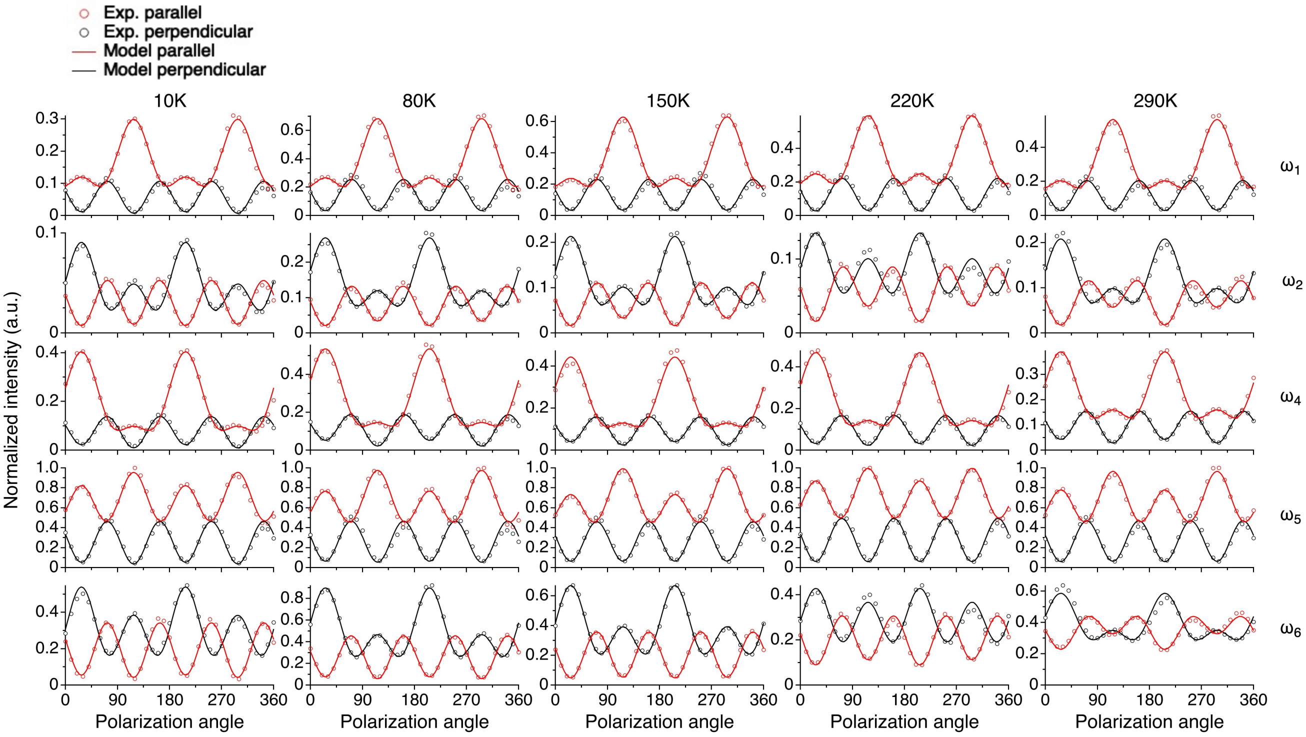

Figure S12: Fitting results for the PO Raman response of anthracene at different temperatures, showing the successful fit obtained by using the fourth-rank formalism.

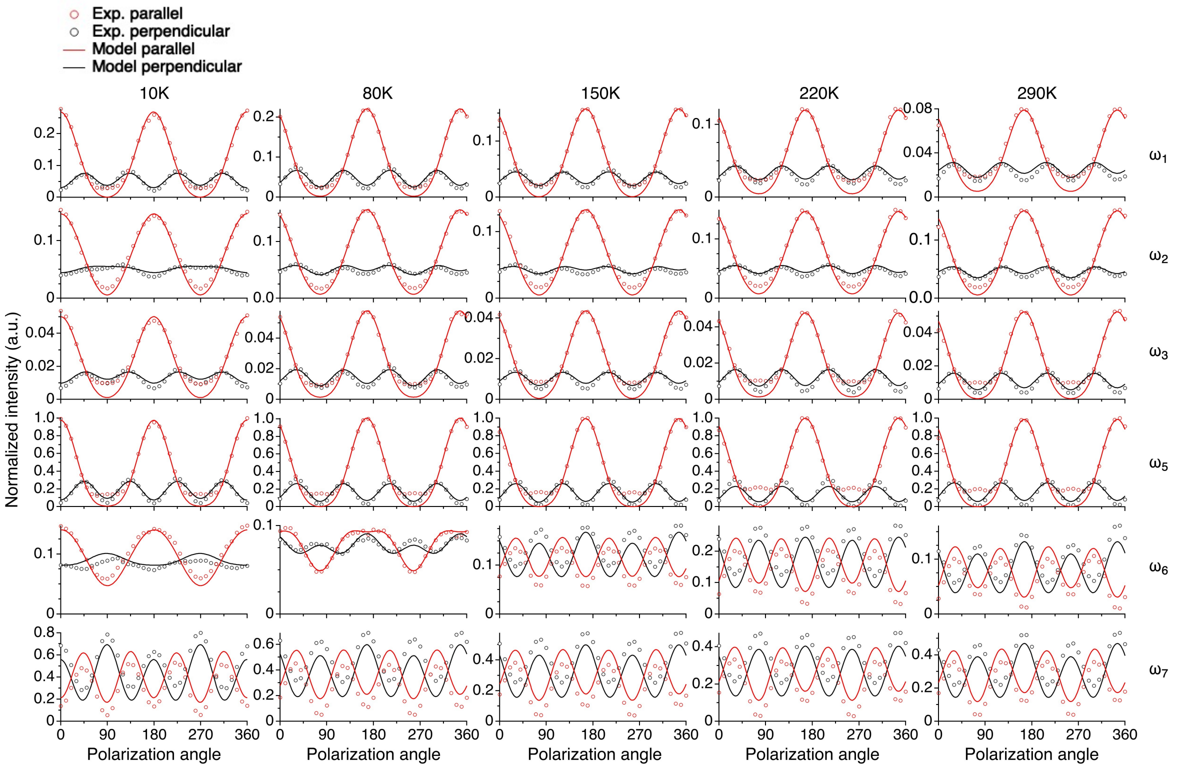

Figure S13: Fitting results for the PO Raman response of pentacene at different temperatures, using a general second-rank Raman tensor. The fit for peaks ω_5, ω_6, ω_7 is unsatisfactory.

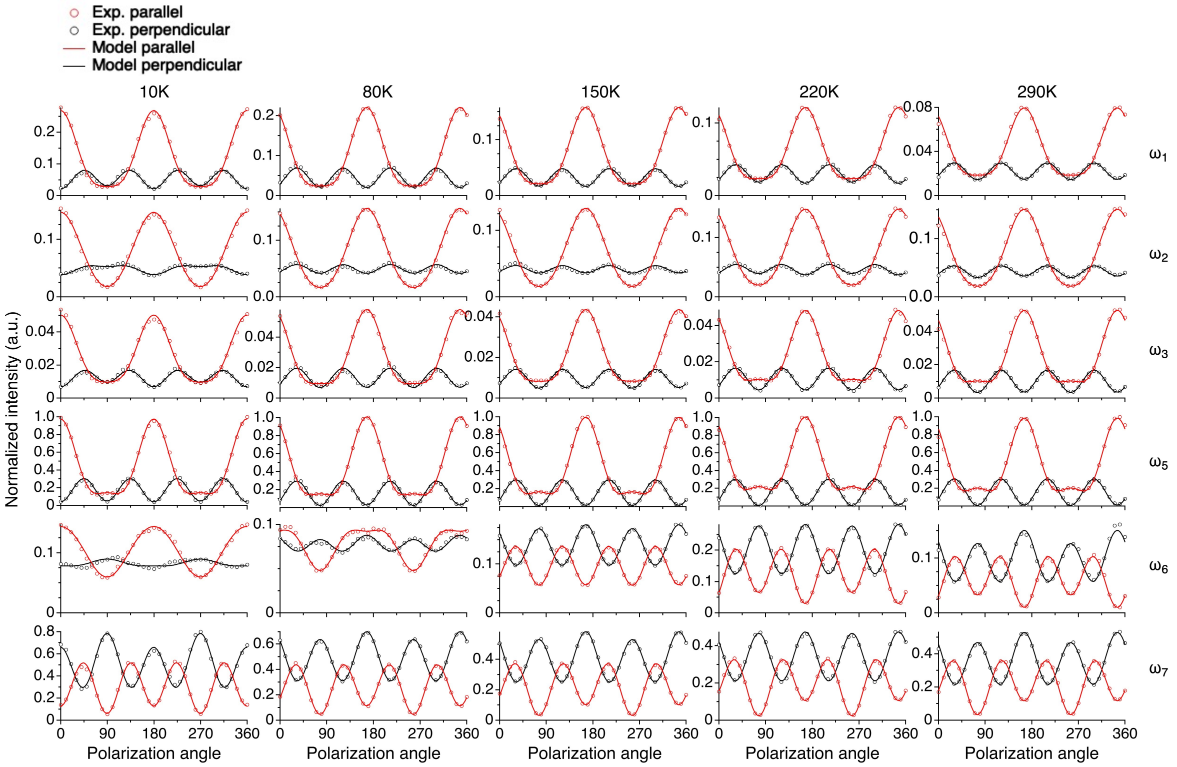

Figure S14: Fitting results for the PO Raman response of pentacene at different temperatures, showing the successful fit obtained by using the fourth-rank formalism.

SXI Theory of inelastic light scattering from crystals at finite temperatures

SXI.1 Vibrational excitations of an anharmonic crystal

In this section we outline the procedure to determine the Raman spectrum of an anharmonic crystal. We will begin by sketching the procedure to obtain the phonon-phonon correlation function in the presence of three-phonon anharmonicity via the equation of motion approach.

If we express the atomic displacements in terms of creation and annihilation operators we can identify the reciprocal space coefficients needed for the perturbative expansion, repeated here for clarity and consistency of notation. Start with the standard creation and annihilation operators for a phonon with momentum $\boldsymbol{q}$ and polarization s (when there is no loss of clarity we use the compound index λ to denote $\boldsymbol{q}s$):

$$a_{\boldsymbol{q}s} = \frac{1}{\sqrt{2N\hbar}} \sum_{i\alpha} \epsilon^{i\alpha}_{\boldsymbol{q}s} \left(\sqrt{m_i \omega_{\boldsymbol{q}s}} u_{i\alpha} - i \frac{p_{i\alpha}}{\sqrt{m_i \omega_{\boldsymbol{q}s}}} \right) e^{-i\boldsymbol{q}\cdot\boldsymbol{r}_i} \tag{S18}$$

$$a^{\dagger}_{\boldsymbol{q}s} = \frac{1}{\sqrt{2N\hbar}} \sum_{i\alpha} \epsilon^{i\alpha\dagger}_{\boldsymbol{q}s} \left(\sqrt{m_i \omega_{\boldsymbol{q}s}} u_{i\alpha} + i \frac{p_{i\alpha}}{\sqrt{m_i \omega_{\boldsymbol{q}s}}} \right) e^{i\boldsymbol{q}\cdot\boldsymbol{r}_i} \tag{S19}$$

$$A_{\boldsymbol{q}s} = a_{\boldsymbol{q}s} + a^{\dagger}_{\bar{\boldsymbol{q}}s} = \frac{2}{\sqrt{2N\hbar}} \sum_{i\alpha} \epsilon^{i\alpha}_{\boldsymbol{q}s} \sqrt{m_i \omega_{\boldsymbol{q}s}} u_{i\alpha} e^{-i\boldsymbol{q}\cdot\boldsymbol{r}_i} \tag{S20}$$

$$B_{\boldsymbol{q}s} = a_{\boldsymbol{q}s} - a^{\dagger}_{\bar{\boldsymbol{q}}s} = - \frac{2i}{\sqrt{2N\hbar}} \sum_{i\alpha} \epsilon^{i\alpha}_{\boldsymbol{q}s} \frac{p_{i\alpha}}{\sqrt{m_i \omega_{\boldsymbol{q}s}}} e^{-i\boldsymbol{q}\cdot\boldsymbol{r}_i} \tag{S21}$$

Here ϵ are phonon eigenvectors ($\boldsymbol{\epsilon}_i \cdot \boldsymbol{\epsilon}_j = \delta_{ij}$), m atomic masses and ω frequencies. The vector $\boldsymbol{r}$ is a lattice vector, and u and p are the position and momentum operators. We use the notation $\bar{\boldsymbol{q}} = -\boldsymbol{q}$. We introduce scaled eigenvectors (to simplify notation) via

$$\upsilon^{i\alpha}_{\boldsymbol{q}s} = \sqrt{\frac{\hbar}{2 m_i \omega_{\boldsymbol{q}s}}} \epsilon^{i\alpha}_{\boldsymbol{q}s} \tag{S22}$$

In this notation the matrix elements pertaining to anharmonicity become

$$\Phi_{\lambda\lambda'\lambda''} = \frac{1}{3!} \sum_{ijk\alpha\beta\gamma} \upsilon^{i\alpha}_{\boldsymbol{q}s} \upsilon^{j\beta}_{\boldsymbol{q}'s'} \upsilon^{k\gamma}_{\boldsymbol{q}''s''} \Phi^{\alpha\beta\gamma}_{ijk} e^{-i(\boldsymbol{q}\cdot\boldsymbol{r}_i + \boldsymbol{q}'\cdot\boldsymbol{r}_j + \boldsymbol{q}''\cdot\boldsymbol{r}_k)} \tag{S23}$$

$$\Phi_{\lambda\lambda'\lambda''\lambda'''} = \frac{1}{4!} \sum_{ijkl\alpha\beta\gamma\delta} \upsilon^{i\alpha}_{\boldsymbol{q}s} \upsilon^{j\beta}_{\boldsymbol{q}'s'} \upsilon^{k\gamma}_{\boldsymbol{q}''s''} \upsilon^{l\delta}_{\boldsymbol{q}'''s'''} \Phi^{\alpha\beta\gamma\delta}_{ijkl} e^{-i(\boldsymbol{q}\cdot\boldsymbol{r}_i + \boldsymbol{q}'\cdot\boldsymbol{r}_j + \boldsymbol{q}''\cdot\boldsymbol{r}_k + \boldsymbol{q}'''\cdot\boldsymbol{r}_l)} \tag{S24}$$

so that the anharmonic part of the Hamiltonian becomes

$$H_A = \Phi_{\lambda\lambda'\lambda''} A_\lambda A_{\lambda'} A_{\lambda''} + \Phi_{\lambda\lambda'\lambda''\lambda'''} A_\lambda A_{\lambda'} A_{\lambda''} A_{\lambda'''} + \cdots . \tag{S25}$$

In an analogous way we define the coefficients of the expansion of the polarizability as

$$\chi^{\mu\nu}_{\lambda} = \sum_{i\alpha} \upsilon^{i\alpha}_{\boldsymbol{q}s} \chi^{\mu\nu,\alpha}_{i} e^{-i\boldsymbol{q}\cdot\boldsymbol{r}_i} \Delta_{\boldsymbol{q}} = \sum_{i\alpha} \upsilon^{i\alpha}_{\boldsymbol{\Gamma}s} \chi^{\mu\nu,\alpha}_{i} = \chi^{\mu\nu}(s) \tag{S26}$$

$$\chi^{\mu\nu}_{\lambda\lambda'} = \frac{1}{2!} \sum_{ij\alpha\beta} \upsilon^{i\alpha}_{\boldsymbol{q}s} \upsilon^{j\beta}_{\boldsymbol{q}'s'} \chi^{\mu\nu,\alpha\beta}_{ij} e^{-i\boldsymbol{q}\cdot\boldsymbol{r}_i} e^{-i\boldsymbol{q}'\cdot\boldsymbol{r}_j} \Delta_{\boldsymbol{q}\boldsymbol{q}'} \tag{S27}$$

$$= \frac{1}{2!} \sum_{ij\alpha\beta} \upsilon^{i\alpha}_{\boldsymbol{q}s} (\upsilon^{j\beta}_{\boldsymbol{q}s'})^\dagger \chi^{\mu\nu,\alpha\beta}_{ij} e^{i\boldsymbol{q}\cdot(\boldsymbol{r}_j - \boldsymbol{r}_i)} \tag{S28}$$

$$= \frac{1}{2!} \sum_{ij\alpha\beta} (\upsilon^{i\alpha}_{\boldsymbol{q}s})^\dagger \upsilon^{j\beta}_{\boldsymbol{q}s'} \chi^{\mu\nu,\alpha\beta}_{ij} e^{-i\boldsymbol{q}\cdot(\boldsymbol{r}_j - \boldsymbol{r}_i)} = \chi^{\mu\nu}(\boldsymbol{q}, s, s') = \chi^{\mu\nu}(\bar{\boldsymbol{q}}, s, s')^\dagger = \chi^{\mu\nu}(\bar{\boldsymbol{q}}, s', s) \tag{S29}$$

$$\chi^{\mu\nu}_{\lambda\lambda'\lambda''} = \frac{1}{3!} \sum_{ijk\alpha\beta\gamma} \upsilon^{i\alpha}_{\boldsymbol{q}s} \upsilon^{j\beta}_{\boldsymbol{q}'s'} \upsilon^{k\gamma}_{\boldsymbol{q}'s'} \chi^{\mu\nu,\alpha\beta\gamma}_{ijk} e^{-i(\boldsymbol{q}\cdot\boldsymbol{r}_i + \boldsymbol{q}'\cdot\boldsymbol{r}_j + \boldsymbol{q}''\cdot\boldsymbol{r}_k)} \tag{S30}$$

Such that

$$\chi^{\mu\nu} = \chi^{\mu\nu}_0 + \chi^{\mu\nu}_\lambda A_\lambda + \chi^{\mu\nu}_{\lambda\lambda'} A_\lambda A_{\lambda'} + \chi^{\mu\nu}_{\lambda\lambda'\lambda''} A_\lambda A_{\lambda'} A_{\lambda''} + \cdots . \tag{S31}$$

SXI.2 Raman scattering in terms of the anharmonic Green's function

If we start from the polarizability-polarizability correlation function and insert the expansion of the polarizability in terms of phonon coordinates, Eq. (S31), we get

$$\begin{aligned}
\langle \chi(t)\chi(0) \rangle =& \boldsymbol{\chi}_0 \otimes \boldsymbol{\chi}_0 + \boldsymbol{\chi}_\lambda \otimes \boldsymbol{\chi}_{\lambda'} i\overset{>}{G}(A_\lambda, A_{\lambda'}) + \\
&+ \boldsymbol{\chi}_{\lambda\lambda'} \otimes \boldsymbol{\chi}_{\lambda''\lambda'''} i\overset{>}{G}(A_\lambda A_{\lambda'}, A_{\lambda''} A_{\lambda'''}) + \\
&+ \boldsymbol{\chi}_\lambda \otimes \boldsymbol{\chi}_{\lambda'\lambda''} i\overset{>}{G}(A_\lambda, A_{\lambda'} A_{\lambda''}) + \boldsymbol{\chi}_{\lambda\lambda'} \otimes \boldsymbol{\chi}_{\lambda''} i\overset{>}{G}(A_\lambda A_{\lambda'}, A_{\lambda''}) + \\
&+ \boldsymbol{\chi}_\lambda \otimes \boldsymbol{\chi}_{\lambda'\lambda''\lambda'''} i\overset{>}{G}(A_\lambda, A_{\lambda'} A_{\lambda''} A_{\lambda'''}) + \boldsymbol{\chi}_{\lambda\lambda'\lambda''} \otimes \boldsymbol{\chi}_{\lambda'''} i\overset{>}{G}(A_\lambda A_{\lambda'} A_{\lambda''}, A_{\lambda'''}),
\end{aligned} \tag{S32}$$

where $\overset{>}{G}(A_\lambda, A^\dagger_{\lambda'}, \tau) \equiv -i\langle A_\lambda(\tau), A_{\lambda'}(0)\rangle$, and we have omitted terms of A^4 or higher. The physical quantity of interest here is the spectral function, $J_{\lambda\lambda'}(\Omega)$, which may also be written in terms of

the retarded Green's function $G^R_{\lambda\lambda'}$:

$$G^R_{\lambda\lambda'}(\Omega, T) = -i\int \theta(\tau)\left\langle\left[A_\lambda(\tau), A^\dagger_{\lambda'}(0)\right]\right\rangle_T e^{-i\Omega t}\mathrm{d}t, \tag{S33}$$

$$J_{\lambda\lambda'}(\Omega) = -\frac{1}{\pi}\mathrm{Im}\left\{G^R_{\lambda\lambda'}(\Omega)\right\} = \frac{i}{(n(\Omega,T)+1)}\overset{>}{G}_{\lambda\lambda'}(\Omega), \tag{S34}$$

where $n(\Omega, T)$ is the Bose-Einstein occupation number for energy $\hbar\Omega$ at temperature T. The retarded Green's function is conveniently given in terms of the sum of a diagonal harmonic part, $g^0_{\lambda\lambda'}(\Omega)$, and the self-energy term $\Sigma_{\lambda\lambda'}(\Omega)$:

$$G^R(\Omega)^{-1} = g^0(\Omega)^{-1} + \Sigma(\Omega). \tag{S35}$$

Following the work of others,[15;16;17;18] we have previously discussed in detail the solution for the retarded Green's function of an anharmonic system H_A.[19] In the case of third-order anharmonicity, the self-energy is given by

$$\Sigma_{\lambda\lambda'}(\Omega) = -18\sum_{\boldsymbol{q}_1\boldsymbol{q}_2 s_1 s_2}\Phi^{\lambda s_1 s_2}_{\boldsymbol{q}\bar{\boldsymbol{q}}_1\bar{\boldsymbol{q}}_2}\Phi^{\lambda' s_1 s_2}_{\bar{\boldsymbol{q}}\boldsymbol{q}_1\boldsymbol{q}_2}S(s_1, s_2, \Omega) \tag{S36}$$

and

$$\begin{aligned} S(s_a, s_b, \Omega) = (n_a + n_b + 1)&\left[\frac{1}{(\omega_a+\omega_b-\Omega)_p} - \frac{1}{(\omega_a+\omega_b+\Omega)_p}\right] \\ &+(n_a - n_b)\left[\frac{1}{(\omega_b-\omega_a+\Omega)_p} - \frac{1}{(\omega_b-\omega_a-\Omega)_p}\right]. \end{aligned} \tag{S37}$$

The constant (zeroth) term in Eq. (S32) does not contribute to the Raman spectrum. Using the spectral function (S33), the first term becomes

$$I^{\mathrm{I}} = \boldsymbol{\chi}_\lambda \otimes \boldsymbol{\chi}_{\lambda'}\int i\overset{>}{G}(A_\lambda(t), A_{\lambda'}(0))e^{-i\Omega t}\mathrm{d}t = \boldsymbol{\chi}_\lambda \otimes \boldsymbol{\chi}_{\lambda'}(n(\Omega)+1)J_{\lambda\lambda'}(\Omega). \tag{S38}$$

This is sufficient to understand the origin of the polarization dependence presented in this study. Higher terms in expansion (S32) are usually smaller, but become significant in Raman inactive structures, where the derivatives in Eq. (S38) identically vanish. This will happen for any crystal where all atomic sites constitute a center of inversion. See[19] for a detailed discussion for the case of rock-salt structure, as well as how higher-order terms might play a role in finite temperatures.

SXI.3 Light scattering in harmonic systems

In harmonic systems all inter-atomic interactions are reduced to terms quadratic in their displacement from equilibrium in the potential expansion.[20] These produce ideal, non-interacting normal mode solutions, $G_{\lambda\lambda'}(\Omega) = g^0_\lambda(\Omega)\delta_{\lambda\lambda'}$.[16] Using the harmonic spectral function,[21] the Stokes component in Eq. (S38) becomes

$$I(\Omega, T) = \sum_\lambda \boldsymbol{\chi}^*_\lambda \otimes \boldsymbol{\chi}_\lambda \left[n(\Omega, T) + 1\right] \delta(\Omega - \omega_\lambda). \tag{S39}$$

The inadequacy of a purely harmonic treatment in light scattering is already partially recognized in most Raman studies, at least implicitly. As Eq. (S39) shows, the frequency dependence of a truly harmonic system should be a delta-function. In practice, a Lorentzian-like line shape is commonly used, and explained by the same spectral function $J(\Omega)$, or more explicitly $(\mathrm{Im}\{\Sigma_{\lambda\lambda}\})^{-1}$, commonly known as the phonon lifetime.[22] In addition, thermal expansion is sometimes incorporated through a quasi-harmonic treatment where the normal modes become volume dependent.[23]

If only diagonal terms are considered (note that the self-energy need not vanish for this), the generalized fourth-rank expression of Eq. (S6) reduces back to the temperature *independent* PO response (Eq. (1) in main text), and all PO patterns may be explained in terms of a single second-rank tensor $\mathcal{R}$. This is demonstrated by plugging Eq. (S39) into Eq. (S6). The incident field, $\boldsymbol{E}_i$, observed scattering polarization, $\boldsymbol{n}$, and second-rank tensor, $\boldsymbol{\chi}_\lambda$ are defined as:

$$\boldsymbol{E}_i = |\boldsymbol{E}_i| \begin{pmatrix} e_x \\ e_y \\ e_z \end{pmatrix}; \quad \boldsymbol{n} \equiv \hat{\boldsymbol{e}}_s = \begin{pmatrix} n_x \\ n_y \\ n_z \end{pmatrix}; \quad \mathcal{R} \equiv \boldsymbol{\chi}_\lambda = \begin{pmatrix} \chi_{xx} & \chi_{xy} & \chi_{xz} \\ \chi_{yx} & \chi_{yy} & \chi_{yz} \\ \chi_{zx} & \chi_{zy} & \chi_{zz} \end{pmatrix}. \tag{S40}$$

The polarization dependence of the scattering cross-section for a single mode λ becomes:

$$
\begin{aligned}
\sigma &\propto \sum_{\mu\nu\xi\rho} n_\mu n_\xi I_{\mu\nu,\xi\rho} E_\nu E_\rho \\
&= [n(\omega_\lambda, T) + 1] \left|\boldsymbol{E}_i\right|^2 \sum_{\mu\nu\xi\rho} n_\mu n_\xi \left(\boldsymbol{\chi}_\lambda^* \otimes \boldsymbol{\chi}_\lambda\right)_{\mu\nu\xi\rho} e_\nu e_\rho \\
&\propto \sum_{\mu\nu\xi\rho} n_\mu n_\xi \chi_{\nu\mu}^* \chi_{\rho\xi} e_\nu e_\rho \\
&= \left(\sum_{\nu\mu} \chi_{\nu\mu}^* n_\mu e_\nu\right) \left(\sum_{\rho\xi} \chi_{\rho\xi} n_\xi e_\rho\right) = \left(\sum_{\nu\mu} \chi_{\nu\mu} n_\mu e_\nu\right)^* \left(\sum_{\rho\xi} \chi_{\rho\xi} n_\xi e_\rho\right) \\
&= \left|\sum_{lm} \chi_{lm} n_m e_l\right|^2 = \left|\sum_l e_l \sum_m \chi_{lm} n_m\right|^2 \\
&= \left|\hat{\boldsymbol{e}}_i \cdot \mathcal{R} \cdot \hat{\boldsymbol{e}}_s\right|^2,
\end{aligned}
$$

which is exactly the original second-rank expression.

References

[1] Vyas, V. S.; Gutzler, R.; Nuss, J.; Kern, K.; Lotsch, B. V. Optical Gap in Herringbone and Π-stacked Crystals of [1]Benzothieno[3,2-b]benzothiophene and its Brominated Derivative. *CrystEngComm* **2014**, *16*, 7389–7392.

[2] Niebel, C.; Kim, Y.; Ruzié, C.; Karpinska, J.; Chattopadhyay, B.; Schweicher, G.; Richard, A.; Lemaur, V.; Olivier, Y.; Cornil, J. et al. Thienoacene Dimers Based on the Thieno[3,2-b]thiophene Moiety: Synthesis, Characterization and Electronic Properties. *Journal of Materials Chemistry C* **2015**, *3*, 674–685.

[3] Matsumura, M.; Muranaka, A.; Kurihara, R.; Kanai, M.; Yoshida, K.; Kakusawa, N.; Hashizume, D.; Uchiyama, M.; Yasuike, S. General Synthesis, Structure, and Optical Properties of Benzothiophene-Fused Benzoheteroles Containing Group 15 and 16 Elements. *Tetrahedron* **2016**, *72*, 8085–8090.

[4] Macrae, C. F.; Bruno, I. J.; Chisholm, J. A.; Edgington, P. R.; McCabe, P.; Pidcock, E.; Rodriguez-Monge, L.; Taylor, R.; Van De Streek, J.; Wood, P. A. Mercury CSD 2.0 - New Features for the Visualization and Investigation of Crystal Structures. *Journal of Applied Crystallography* **2008**, *41*, 466–470.

[5] Macrae, C. F.; Edgington, P. R.; McCabe, P.; Pidcock, E.; Shields, G. P.; Taylor, R.; Towler, M.; Van De Streek, J. Mercury: Visualization and Analysis of Crystal Structures. *Journal of Applied Crystallography* **2006**, *39*, 453–457.

[6] Lu, Z. Q.; Quinn, T.; Reehal, H. S. Polarization-Dependent Raman Spectra of Thin Crystalline Silicon Films. *Journal of Applied Physics* **2005**, *97*, 033512.

[7] Rousseau, D. L.; Bauman, R. P.; Porto, S. P. S. Normal Mode Determination in Crystals. *Journal of Raman Spectroscopy* **1981**, *10*, 253–290.

[8] Aroyo, M. I.; Perez-Mato, J. M.; Capillas, C.; Kroumova, E.; Ivantchev, S.; Madariaga, G.; Kirov, A.; Wondratschek, H. Bilbao Crystallographic Server: I. Databases and Crystallographic Computing Programs. *Zeitschrift für Kristallographie - Crystalline Materials* **2006**, *221*, 15–27.

[9] Asher, M.; Angerer, D.; Korobko, R.; Diskin-Posner, Y.; Egger, D. A.; Yaffe, O. Anharmonic Lattice Vibrations in Small-molecule Organic Semiconductors. *Advanced Materials* **2020**, *32*, 1908028.

[10] Kranert, C.; Sturm, C.; Schmidt-Grund, R.; Grundmann, M. Raman Tensor Formalism for Optically Anisotropic Crystals. *Physical Review Letters* **2016**, *116*, 127401.

[11] Yu, P. Y.; Cardona, M. *Fundamentals of Semiconductors*; Graduate Texts in Physics; Springer Berlin Heidelberg: Berlin, Heidelberg, 2010; Vol. 1.

[12] Nye, J.; Nye, P. *Physical Properties of Crystals: Their Representation by Tensors and Matrices*; Oxford science publications; Clarendon Press, 1985.

[13] Slezák, O.; Lucianetti, A.; Mocek, T. Tensor-to-Matrix Mapping in Elasto-Optics. *J. Opt. Soc. Am. B* **2020**, *37*, 1090–1097.

[14] van den Bos, A. *Parameter Estimation for Scientists and Engineers*; John Wiley & Sons, Inc.: Hoboken, NJ, USA, 2007; pp 259–263.

[15] Leibfried, G.; Ludwig, W. Theory of Anharmonic Effects in Crystals. *Solid State Physics - Advances in Research and Applications* **1961**, *12*, 275–444.

[16] Cowley, R. The Lattice Dynamics of an Anharmonic Crystal. *Advances in Physics* **1963**, *12*, 421–480.

[17] Wallace, D. C. *Thermodynamics of Crystals*; Dover Books on Physics; Dover Publications, Incorporated, 1998.

[18] Semwal, B. S.; Sharma, P. K. Thermal Conductivity of an Anharmonic Crystal. *Physical Review B* **1972**, *5*, 3909–3914.

[19] Benshalom, N.; Reuveni, G.; Korobko, R.; Yaffe, O.; Hellman, O. Dielectric Response of Rock-Salt Crystals at Finite Temperatures From First Principles. *Physical Review Materials* **2022**, *6*, 033607.

[20] Dove, M. *Structure and Dynamics: An Atomic View of Materials*, 1st ed.; Oxford University Press Inc.: New-York, 2003; p 334.

[21] Kwok, P. C. In *Green's Function Method in Lattice Dynamics*; Seitz, F., Turnbull, D., Ehrenreich, H. B. T. S. S. P., Eds.; Academic Press, 1968; Vol. 20; pp 213–303.

[22] Maradudin, A. A.; Fein, A. E.; Vineyard, G. H. On the Evaluation of Phonon Widths and Shifts. *Physica Status Solidi (B)* **1962**, *2*, 1479–1492.

[23] Fultz, B. Vibrational Thermodynamics of Materials. *Progress in Materials Science* **2010**, *55*, 247–352.
